# Lotus germ extract rejuvenates aging fibroblasts via restoration of disrupted proteostasis by the induction of autophagy

**DOI:** 10.18632/aging.204303

**Published:** 2022-09-26

**Authors:** Kayo Machihara, Sou Kageyama, Shoma Oki, Hiroki Makino, Masamichi Sasaki, Hiroyasu Iwahashi, Takushi Namba

**Affiliations:** 1 Research and Education Faculty, Multidisciplinary Science Cluster, Interdisciplinary Science Unit, Kochi University, Kochi 783-8505, Japan; 2 Department of Marine Resource Science, Faculty of Agriculture and Marine Science, Kochi University, Kochi 783-8502, Japan; 3 Research Center, Maruzen Pharmaceuticals Co. Ltd., Fukuyama City, Hiroshima 729-3102, Japan

**Keywords:** mitochondria, autophagy, proteostasis, aging

## Abstract

Cell aging attenuates cellular functions, resulting in time-dependent disruption of cellular homeostasis, which maintains the functions of proteins and organelles. Mitochondria are important organelles responsible for cellular energy production and various metabolic processes, and their dysfunction is strongly related to the progression of cellular aging. Here we demonstrate that disruption of proteostasis attenuates mitochondrial function before the induction of DNA damage signaling by proliferative and replicative cellular aging. We found that lotus (*Nelumbo nucifera* Gaertn.) germ extract clears abnormal proteins and agglutinates via autophagy-mediated restoration of mitochondrial function and cellular aging phenotypes. Pharmacological analyses revealed that DAPK1 expression was suppressed in aging cells, and lotus germ extract upregulated DAPK1 expression by stimulating the acetylation of histones and then induced autophagy by activating the DAPK1-Beclin1 signaling pathway. Furthermore, treatment of aging fibroblasts with lotus germ extract stimulated collagen production and increased contractile ability in three-dimensional cell culture. Thus, time-dependent accumulation of abnormal proteins and agglutinates suppressed mitochondrial function in cells in the early stage of aging, and reactivation of mitochondrial function by restoring proteostasis rejuvenated aging cells. Lotus germ extract rejuvenates aging fibroblasts via the DAPK1-Beclin1 pathway-induced autophagy to clear abnormal proteins and agglutinates.

## INTRODUCTION

Aging is a complex multifunctional biological process that results in time-dependent attenuation of biological function and simultaneously induces the loss of various cellular functions [1]. Aging is an important factor in the onset and exacerbation of various diseases; however, though the molecular mechanisms of its progression have been elucidated, a comprehensive understanding has yet to be reported [1, 2].

Cellular senescence refers to the irreversible loss of cell division capacity due to DNA damage or telomere deletion caused by repeated cell division and several stressors [3]. Aging normal organs contain some senescent cells, and the removal of these senescent cells was found to restore organ function and extend life span in aged mice, indicating that cellular senescence induces whole-tissue functional decline [4, 5]. Thus, suppressing the progression of cellular aging prevents aging-dependent organ dysfunction. Cells lose their original functions when their homeostasis is disrupted due to repeated proliferation and several stressors, leading to various time-dependent effects, such as the production of aging-dependent advanced glycation end products (AGEs), protein aggregation, epigenetic changes, the loss of organelle function, the induction of DNA damage signals, and cell cycle arrest. [1, 3, 6]. Finally, senescent cells stop proliferating and produce senescence-associated secretory phenotype (SASP)-related factors, which impair the function of surrounding cells [3]. However, the sequence of aging-related changes and how they affect each other remain unclear.

Mitochondria play a crucial role in the aging process [7]. Furthermore, mitochondria are one of the most important organelles for maintaining cellular homeostasis, which is involved in ATP production and several metabolic pathways, and mitochondrial function is disrupted over time [8]. Several reports have indicated that damaged mitochondria exhibit decreased function due to their own production of reactive oxygen species (ROS) and abnormal proteins, which are generated by the loss of cellular proteostasis, accelerating the cellular aging process [7, 9, 10]. While reactivation of mitochondrial function can inhibit the aging process, there is also the concern that excessive mitochondrial activation might exacerbate aging by increasing ROS production. Thus, many studies determine at what point in the cellular aging process mitochondrial function declines and how mitochondria can be reactivated during the aging process to suppress aging are still being conducted.

Proteostasis is assured through the coordinated action of intricate cellular systems, such as chaperones, and two proteolytic systems, the ubiquitin-proteasome and autophagy systems. Many species have gradually lost proteostasis with healthy aging, and autophagic function also declines [11]. It has been reported that induction of autophagy, which is reduced by aging, restores proteostasis, and suppresses the aging phenotypes, suggesting that keeping autophagic activity is essential for maintaining proteostasis during aging [12]. Proteostasis disruption has been reported to induce mitochondrial dysfunction. Thus, maintaining proteostasis is also critical for proper mitochondrial function [13].

This study reports that disruption of proteostasis attenuated mitochondrial function upon proliferative and replicative senescence before the induction of DNA damage signaling, and the clearance of abnormal substances via autophagy restored mitochondrial function in aging fibroblasts, which is an early stage of cellular senescence. We screened 75 plant extracts that restored mitochondrial function in aging fibroblasts and discovered that lotus (*Nelumbo nucifera* Gaertn.) germ extract reduced the activity of senescence-associated β-galactosidase (SA-β-gal), a marker of aging, and activated mitochondria via induction of autophagy, which degraded lipofuscin aggregates and AGEs, which increased the aging process. Analysis of the molecular mechanism of lotus germ extract indicated that it induced autophagic signaling; upregulated DAPK1 expression, which is downregulated in an aging-dependent manner; stimulated the phosphorylation of beclin1; and then induced autophagy. We also found that aging-dependent histone acetylation and methylation are related to the suppression of DAPK1 expression and that lotus germ extract causes epigenetic changes close to those observed in youth. Moreover, the treatment of aging fibroblasts with lotus germ extract stimulated collagen production and contractile ability in three-dimensional cell culture. Thus, the induction of noncanonical autophagic signaling via DAPK1 upregulation is a new antiaging target to restore mitochondrial function by the restoration of disturbed proteostasis in the early stages of cellular senescence. Additionally, lotus germ extract was found to rejuvenate aging fibroblasts and is thus a promising new antiaging material.

## RESULTS

### Mitochondrial dysfunction occurs before the induction of DNA damage signaling in proliferative and replicative senescence

**Figure 1:**
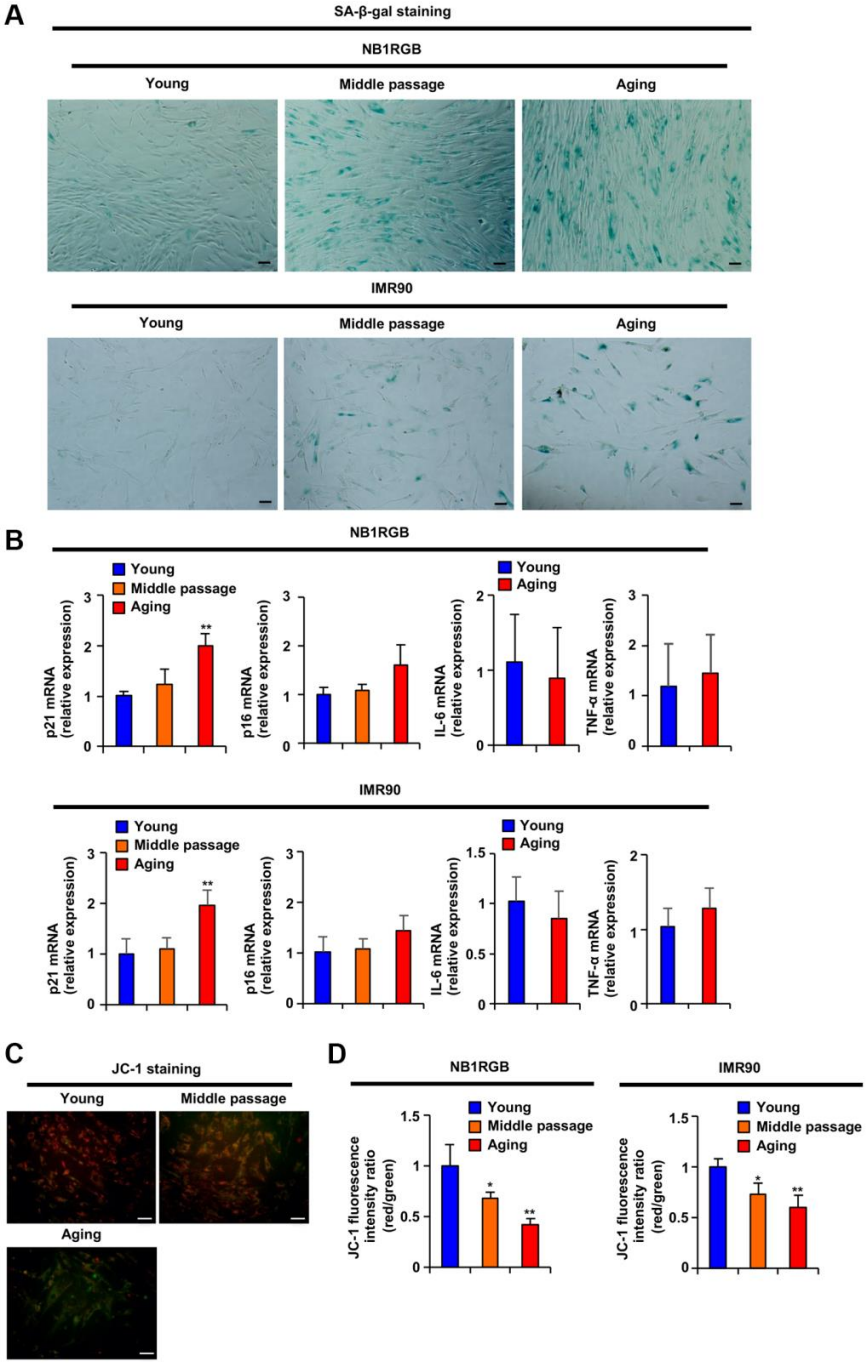
**Replicative aging induced mitochondrial dysfunction.** (**A**) Replicative aging activates SA-β-gal. The indicated cells at several passages were stained as SA-β-gal-positive cells (scale bar, 50 μm). (**B**) Replicative aging induced a genetic aging phenotype. Total RNA was extracted from NB1RGB and IMR90 cells at several passages and subjected to real-time quantitative PCR. Data are presented as the mean ± standard deviation (SD) (three different data sets). (**C** and **D**) Replicative aging decreased mitochondrial transmembrane electric potential. The cells were incubated with JC-1 for 20 min and assessed with (**C**) fluorescence microscopy (red, JC-1 aggregates; green, JC-1 monomers) or (**D**) microplate reader; ΔΨ*m* was determined by the ratio between the red fluorescence intensity, indicating activated mitochondria, and the green fluorescence intensity, indicating deactivated mitochondria. The data are presented as the mean ± SD of three simultaneously performed experiments (**B**, **D**). Each *P* value was calculated using two-way ANOVA; ^*^P < 0.05, ^**^P < 0.01.

NB1RGB and IMR90 cells, which are human diploid fibroblasts (HDFs), at different stages of proliferative and replicative aging showed several aging-related markers, such as Senescence associated-β-galactosidase (SA-β-gal) and genetic markers (DNA damage signal: p21 and p16, SASP factors: IL-6 and TNF-α), as shown in Figure 1A and 1B. Previously, p21 expression and SA-β-gal activity were reported to be increased in early senescence, and p16 and SASP factor gene expression was found to be induced in late senescence [14]. As shown in Figure 1A and 1B, aging fibroblasts exhibited SA-β-gal activity and increased p21 mRNA levels, but p16, IL-6 and TNF-α mRNA expression was no different than that in young fibroblasts, indicating that our established aging fibroblasts were in the early stage of senescence but not fully senescent. Mitochondrial transmembrane potential (ΔΨ*m*) reflects mitochondrial function. We analyzed the effect of these HDFs on ΔΨ*m* using the fluorescent probe JC-1. Red fluorescence shows JC-1 aggregates that appear in the mitochondria after potential-dependent aggregation. Green fluorescence shows JC-1 monomers that appear in the cytosol after mitochondrial membrane depolarization. Aging and middle-passage fibroblasts exhibited decreased ΔΨ*m*, as demonstrated by analysis with fluorescence microscopy (Figure 1C) and microplate reader (Figure 1D). These results suggested that mitochondrial dysfunction and increased SA-β-gal activity were induced earlier than the upregulation of p21 mRNA expression and that aging fibroblasts were in the early phase of cellular senescence.

### Lotus germ extract reactivated mitochondrial function in aging fibroblasts

**Figure 2:**
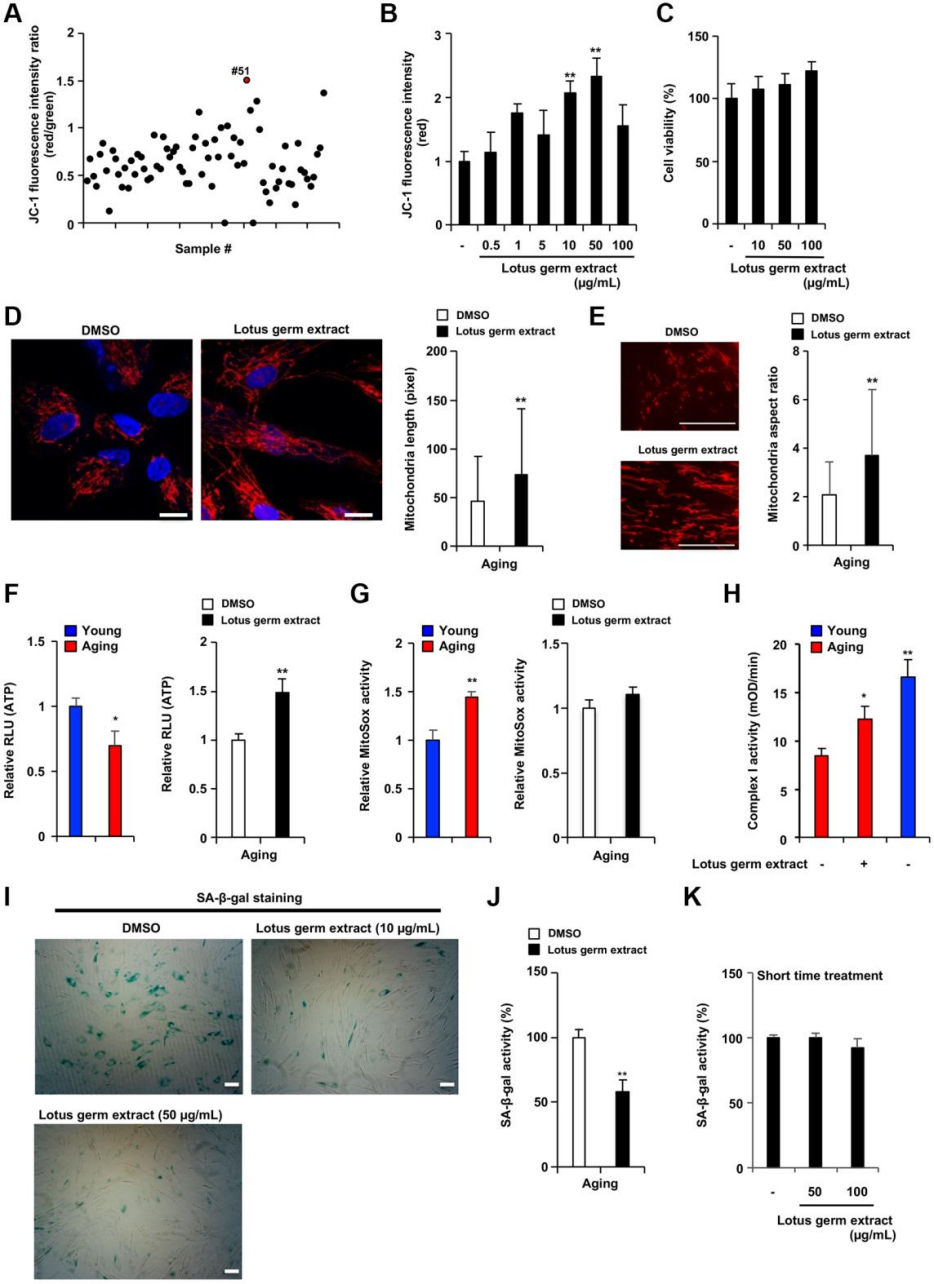
**Lotus germ extract restored mitochondrial function and suppressed the aging phenotype.** (**A**) Plant extract screening was performed in duplicate, and relative JC-1 activity was calculated using the procedure described in Figure 1C. (**B** and **C**) Effects of lotus germ extract on JC-1 activity and cell viability. Aging NB1RGB cells were treated with DMSO (−) or lotus germ extract at the indicated concentration for 24 h. (**B**) JC-1 activity was determined by the red fluorescence intensity, indicating activated mitochondria. (**C**) Cell viability was determined using an MTT assay. (**D** and **E**) Lotus germ extract induced mitochondrial morphological changes. Aging NB1RGB cells were treated with DMSO or 50 μg/mL lotus germ extract for 2 days, followed by treatment with MitoTracker Orange and fluorescence microscopy. Red represents mitochondrial MitoTracker staining, and blue represents nuclear DAPI staining (scale bar, 20 μm). (**D**) The length of the mitochondria within the cells was determined, and the data are presented as the mean ± SD (*n* = 30) (right panel). (**E**) The aspect ratio of the mitochondria within the cells was determined. The data are presented as the mean ± SD (DMSO: *n* = 83, lotus germ extract: *n* = 53) (right panel). (**F** and **G**) Lotus germ extract stimulated ATP but not ROS production. Young and aging cell were compared (left panel). Aging NB1RGB cells were treated with DMSO or 50 μg/mL lotus germ extract for 24 h (right panel). (**F**) ATP levels were determined using a CellTiter-Glo assay. (**G**) The ROS level was determined MitoSOX staining assay. (**H**) Mitochondrial complex I activity is stimulated by the lotus germ extract. Young NB1RGB cells treated with DMSO (−) or NB1RGB cells treated with DMSO (−) or 50 μg/mL of lotus germ extract (+) for 24 h were subsequently assessed for mitochondrial respiratory complex I activity. mOD, millioptical density. (**I**) Lotus germ extract decreased SA-β-gal-positive cells. Aging NB1RGB cells were treated with the indicated concentration of lotus germ extract for 3 days and stained to detect SA-β-gal-positive cells. (**J** and **K**) Lotus germ extract decreased SA-β-gal expression but did not directly suppress SA-β-gal activity. (**J**) Aging NB1RGB cells were treated with the indicated concentration of lotus germ extract for 3 days, and SA-β-gal activity was measured. (**K**) Aging NB1RGB cells were treated with DMSO (−) or the indicated concentration of the lotus germ extract for 2 h. Then, the SA-β-gal activity was measured. Data are presented as the mean ± SD of three simultaneously performed experiments (**B**–**H**, **J** and **K**). Each *P* value was calculated using two-way ANOVA; ^*^P < 0.05, ^**^P < 0.01.

Several reports have suggested that mitochondrial dysfunction stimulates cellular senescence [7, 8]. Our results indicated that mitochondrial dysfunction occurred earlier than the induction of DNA damage signaling and SASP-related gene expression in the proliferative and replicative senescence steps, suggesting that reactivation of mitochondrial function suppressed the progression of cellular senescence in the early stage. We then screened 75 plant extracts (Table 1) to identify those that increase the ΔΨ*m*. As shown in Figure 2A, among these, lotus germ extract (#51) increased the ΔΨ*m* the most in aging fibroblasts, and this increase was dose-dependent without the induction of cell death (Figure 2B, 2C, Supplementary Figures 1A, 1B and 2A upper panel). We confirmed the measurement of ΔΨ*m* activity using a different method as TMRM staining, and we analyzed the dye distribution between mitochondria and the nucleus. As shown in Supplementary Figure 2A (under panel) and Supplementary Figure 2B, the results of the fluorescence image of TMRM staining and the analysis of the distribution of the TMRM fluorescence intensity suggested that lotus germ extract increased TMRM fluorescence intensity. Such an increase was dose-dependent in aging fibroblasts. The analysis result of TMRM fluorescence intensity between mitochondria and the nucleus and microplate reader was very similar (Supplementary Figure 2B and 2C). Thus, we also used microplate reader analysis following TMRM experiments. Lotus germ extract has several bioactive effects, such as its effects against cancer and obesity; however, the effect of lotus germ extract on mitochondrial function has not been elucidated [15]. Previously, it was reported that cycles of fusion and fission modify the morphology of the mitochondrial compartment. Fragmented, short mitochondria (low aspect ratio) have low respiratory activity than fused, long mitochondria (high aspect ratio) [16]. As shown in Figure 2D, we performed imaging analyses using MitoTracker to visualize the mitochondrial morphology, which indicated many short mitochondrial forms in the aging cells (DMSO); however, treatment with lotus germ extract increased the abundance of the long form. The lotus germ extract treatment also increased the aspect ratio of mitochondria in aging fibroblast cells (Figure 2E). ATP production is related to mitochondrial function. A luciferase assay revealed that ATP production was decreased in aging fibroblasts compared to young fibroblasts. The treatment with lotus germ extract significantly increased ATP in aging fibroblasts (Figure 2F and Supplementary Figure 1C). On the other hand, ROS production increased in our established aging fibroblasts compared to young fibroblasts. The lotus germ extract did not increase mitochondrial ROS production in aging fibroblasts, as shown with MitoSOX imaging (Figure 2G). These results suggested that lotus germ extract reactivates mitochondrial function without inducing ROS production. We examined mitochondrial complex I—reduced form of nicotinamide adenine dinucleotide (NADH) dehydrogenase activity—which was measured via spectrophotometric assessment. We found that aging fibroblasts suppressed mitochondrial complex I activity compared young fibroblasts and that lotus germ extract stimulated mitochondrial complex I activity in aging fibroblasts (Figure 2H).

**Table 1:** List of plant extracts.

**No.**	**Scientific name**	**Part of plant**
1	*Vitis vinifera* L.	Leaf
2	*Angelica keiskei* (Miq.) Koidz.	Leaf/Stem
3	*Uncaria gambir* (W. Hunter) Roxb.	Leaf/Scion
4	*Arnica montana* L.	Flower
5	*Ginkgo biloba* L.	Leaf
6	*Urtica dioica* L.	Leaf
7	*Artemisia capillaris* Thunb.	Flower
8	*Curcuma longa* L.	Rhizome
9	*Malva sylvestris* L.	Flower
10	*Camellia sinensis* (L.) Kuntze	Leaf
11	*Rosa multiflora* Thunb.	Fruit
12	*lsodonis japonicus* (Burm.f.) H. Hara	Leaf/Stalk
13	*Scutellaria baicalensis* Georgi	Root
14	*Phellodendron amurense* Rupr.	Bark
15	*Foeniculum vulgare* Mill.	Fruit
16	*Ononis spinosa* *L*.	Root
17	*Olea europaea* L.	Leaf
18	*Artemisia indica Willd.* var. *maximowiczii* (Nakai) H. Hara	Leaf
19	*Matricaria chamomila* L.	Flower
20	*Glycyrrhiza glabra* L.	Root
21	*Glycyrrhiza glabra* *L*.	Leaf
22	*Rubus idaeus* L.	Fruit
23	*Lonicera japonica* Thunb.	Flower
24	*Sophora flavescens* Aiton	Root
25	*Macroptilium atropurpureum* (DC.) Urb.	Flower/Leaf/Stem
26	*Alpinia speciosa* (Wendl.) K. Schum.	Leaf
27	*Gentiana lutea* L.	Rhizome/Root
28	*Oryza sativa* L.	Bran
29	*Punica granatum* L.	Pericarp
30	*Salvia officinalis* L.	Leaf
31	*Crataegus cuneata* Siebold & Zucc.	Fruit
32	*Gardenia jasminoides* J. Ellis	Fruit
33	*Rehmannia glutinosa* Libosch. var. *purpurea* Makino	Root
34	*Perilla frutescens* (L.) Brijton var. *crispa* (Benth.)	Leaf
35	*Paeonia lactiflora* Pall.	Root
36	*Sanguisorba officinalis* L.	Root
37	*Houttuynia cordata* Thunb.	Above-ground part
38	*Averrhoa carambola* L.	Leaf
39	*Centella asiatica* (L.) Urb.	Leaf/Stem
40	*Morus alba* L.	Root
41	*Ziziphus jujuba* Mill. var. *inermis* (Bunge) Rehder	Fruit
42	*Citrus unshiu* (Swingle) Marcow.	Peel
43	*Rubus chingii* Hu var. *suavissimus* (S.K. Lee) L.T. Lu	Leaf
44	*Rhodomyrtus tomentosa* Wight	Fruit
45	*Angelica acutiloba* (Siebold & Zucc.) Kitag.	Root
46	*Calendula officinalis* L.	Flower
47	*Rosa roxburghii* Tratt.	Fruit
48	*Potentilla erecta* (L.) Raeusch.	Root
49	*Panax ginseng* C.A. Mey.	Root
50	*Ananas sativus* Schult. & Schult.f.	Fruit
51	*Nelumbo nucifera* Gaertn.	Germ
52	*Petroselinum crispum* (Mill.) Fuss	Leaf
53	*Hamamelis virginiana* L.	Leaf
54	*Rosa* × *centifolia* L.	Flower
55	*Eriobotrya japonica* (Thunb.) Lidl.	Leaf
56	*Tussilago farfara* L.	Flower
57	*Carthamus tinctorius* L.	Flower
58	*Mentha* × *piperita* L.	Leaf
59	*Paeonia suffruticosa* Andrews	Root
60	*Origanum majorana* L.	Leaf
61	*Actinidia polygama* (Siebold & Zicc.) Planch. ex Maxim.	Fruit
62	*Aesculus hippocastanum* L.	Seed
63	*Acer palmatum* Thunb.	Leaf
64	*Prunus persica* (L.) Batsch	Leaf
65	*Centaurea cyanus* L.	Flower
66	*Saxifraga stolonifera* Curtis	Whole plant
67	*Eucalyptus globulus* Labill.	Leaf
68	*Lilium candidum* L.	Bulb
69	*Coix lacryma-jobi* L. var. *ma-yuen* (Rom.Caill.) Stapf	Seed
70	*Lavandula angustifolia* Mill.	Flower
71	*Melissa* *officinalis* L.	Leaf
72	*Rosmarinus officinalis* L.	Leaf
73	*Thymus serpyllum* L.	Above-ground part
74	*Prunus mume* Siebold & Zucc.	Fruit
75	*Morinda citrifolia* L.	Fruit

Next, we examined whether lotus germ extract affects SA-β-gal activity in aging fibroblasts. As shown in Figure 2I and Supplementary Figure 1D, the number of SA-β-gal-positive cells was decreased by treatment with lotus germ extract in a dose-dependent manner. There are two possible explanations: lotus germ extract inhibits SA-β-gal activity directly or indirectly. As shown in Figure 2J and 2K, aging cells were treated with lotus germ extract for 72 h (j) or 3 h (k). Then, the cells were lysed, and SA-β-gal activity was measured. It was shown in the results that SA-β-gal activity was inhibited by the lotus germ extract in the 72 h treatment but not in the 3 h treatment. Aging fibroblasts were lysed. When the SA-β-gal activity was measured upon adding the lotus germ extract or PETG, the SA-β-gal inhibitor did not inhibit the activity by lotus germ extract even though PETG inhibited SA-β-gal activity (Supplementary Figure 2D). These results suggest that the lotus germ extract suppresses the aging phenotype by altering cellular functions.

### Lotus germ extract induces autophagy and degrades aging-dependent increased lipofuscin-like particles

**Figure 3:**
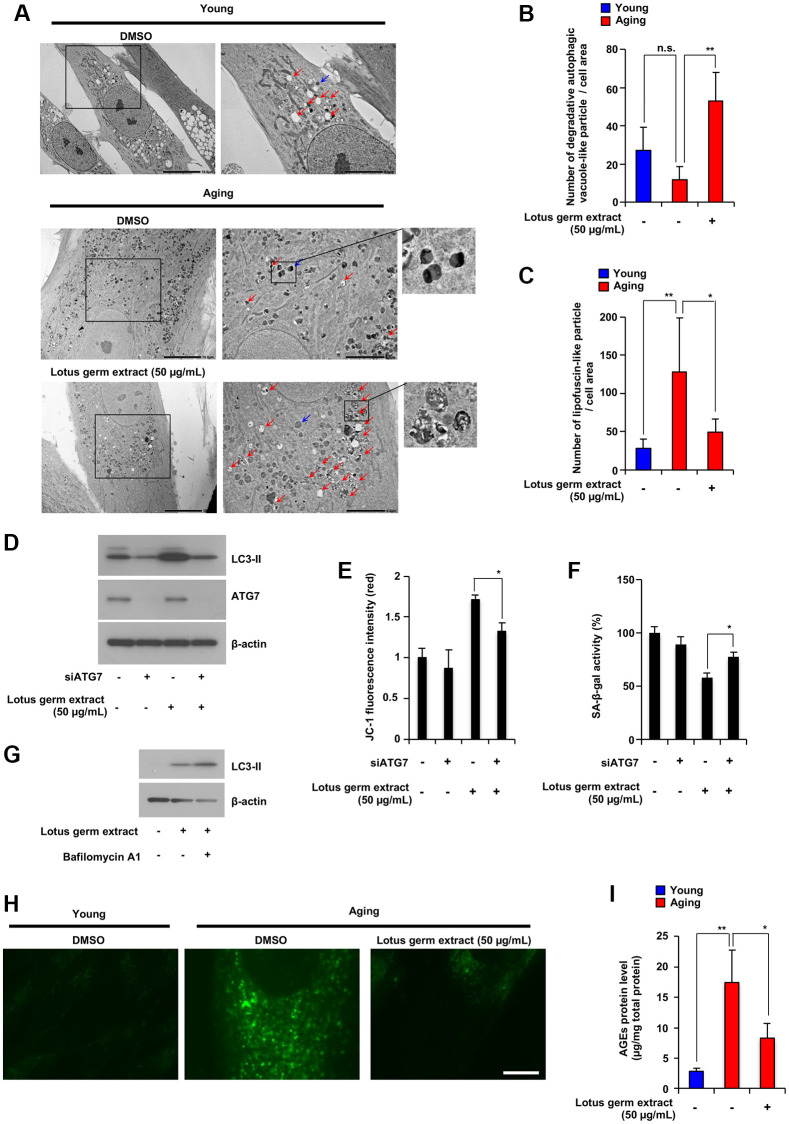
**Lotus germ extract induced autophagy and decreased aging-related accumulation of lipofuscin-like particles and AGEs.** (**A**–**C**) Young and aging NB1RGB cells with DMSO (−) or lotus germ extract (50 μg/mL) treatment (+) were subjected to TEM. The scale bar in the left panel represents 10 μm. Higher magnification images are shown in the right panel, with the scale bar representing 5 μm. Blue arrows indicate aged-related increased lipofuscin-like particles. Red arrows indicate degradative autophagic vacuole-like particles. The numbers of lipofuscin-like particles (**B**) and degradative autophagic vacuole-like particles (**C**) in each cell area were determined. Data are presented as the mean ± SD (*n* = 5). (**D** and **E**) Lotus germ extract induced autophagy, which plays an important role in the suppression of cellular aging phenotypes. Aging NB1RGB cells were transfected with siControl or siATG7 for 24 h, followed by treatment with DMSO (−) or 50 μg/mL lotus germ extract (+) for 24 h. Cells were subjected to immunoblotting using the indicated antibodies (**D**). JC-1 activity was determined based on fluorescence intensity (**E**). (**F**) Aging NB1RGB cells were transfected with siControl or siATG7 for 24 h, followed by treatment with DMSO (−) or 50 μg/mL lotus germ extract (+) for 3 days, and SA-β-gal activity was measured. (**G**) Treatment with the lotus germ extract stimulates autophagosome synthesis. Aging NB1RGB cells were treated with DMSO (−) or 50 μg/mL lotus germ extract (+) for 24 h, followed by treatment with or without bafilomycin A1 (1 μg/mL) (+) for 2 h. Cells were subjected to immunoblotting using the indicated antibodies. (**H** and **I**) Lipofuscin-like particle and AGE levels were decreased by lotus germ extract treatment. Young and aging NB1RGB cells were treated with DMSO (−) or 50 μg/mL lotus germ extract (+) for 3 days and subjected to fluorescence microscopy analysis to detect autofluorescence due to lipofuscin-like particles (**H**) or AGE levels by ELISA (**I**). Data are presented as the mean ± SD of three simultaneously performed experiments (**E** and **F**, **I**). *P* value was calculated using two-way ANOVA; n.s.: not significant, ^*^P < 0.05, ^**^P < 0.01.

To further understand the effect of lotus germ extract on cellular functions, we performed transmission electron microscopy (TEM) to identify any change in the cellular or organelle structure. As shown in Figure 3A, subsequent TEM indicated that lotus germ extract treatment induced the formation of several vacuoles in aging fibroblasts. According to the literature [17], autophagic vacuoles exist in two types, which are initial autophagic vacuoles (AVi) and degradative autophagic vacuoles/autolysosome (AVd). AVis typically have a double membrane, which is usually distinctly visible by TEM as two parallel membrane layers. AVds typically have only one limiting membrane and contain electron-dense cytoplasmic material and/or organelles at various stages of degradation. As shown in Figure 3A under the right panel (lotus germ extract treatment), these vacuoles included electron-dense cytoplasmic material at several stages of degradation and had one limiting membrane. Thus, we considered these vesicles were degradative autophagic-like vacuoles. The treatment of lotus germ extract increased degradative autophagic-like vacuoles in aging fibroblasts (Figure 3A and 3B). In Figure 3A, in the middle right panel, we observed increased lipofuscin-like particles, as shown in the literature [18], in the cytosolic region compared to the abundance of the particles in young fibroblasts (Figure 3A and 3C).

Autophagy is an important cellular response that maintains proteostasis by the bulk degradation of abnormal proteins and organelles that have lost their function. Previously, autophagic dysfunction was reported to stimulate cellular aging, and the induction of autophagy suppresses senescence phenotypes such as the reactivation of mitochondrial function [19]. We further examined whether lotus germ extract treatment affects autophagic induction and whether induced autophagy is related to the reactivation of mitochondria and suppression of the aging phenotype. Aging fibroblasts were treated with or without siRNA for ATG7 and/or lotus germ extract, and levels of the autophagy marker LC3-II were monitored. As shown in Figure 3D, treatment with lotus germ extract increased LC3-II protein expression, and ATG7 knockdown by siRNA silencing suppressed lotus germ extract-induced LC3-II protein expression. ATG7 knockdown significantly suppressed lotus germ extract-induced upregulation of ΔΨ*m* and SA-β-gal in aging fibroblasts (Figure 3E and 3F). I applied bafilomycin A1, which inhibits the degradation of autolysosome contents, to interpret these changes in processed LC3-II levels. Increasing LC3-II protein levels could provide evidence of efficient autophagic flux in the presence of bafilomycin A1. As shown in Figure 3G, co-treatment with the lotus germ extract and bafilomycin A1 increased LC3-II protein levels compared with the treatment with the lotus germ extract alone. This suggests that the lotus germ extract treatment induced autophagosome synthesis. We examined the effect of lotus germ extract treatment on mitophagy and did not find that it induced mitophagy (Supplementary Figure 2E). These results indicate that lotus germ extract-induced autophagy plays an important role in the reactivation of mitochondria, but it might be due to the degradation of abnormal intracellular substances rather than the specific clearance of dysfunctional mitochondria by the induction of mitophagy.

Several reports indicated that lipofuscin is a fluorescent complex mixture composed of highly oxidized cross-linked protein residues, lipids, and sugars, and increased in senescent cells, and age-related lipofuscin aggregates included AGEs, which have also autofluorescence [20]. As shown in Figure 3A and 3C, lotus germ extract decreased age-related lipofuscin-like particles incorporated into autolysosomes. Thus, we confirmed whether autofluorescence and AGE protein levels would be increased in aging fibroblasts and decreased by lotus germ extract treatment. As shown in Figure 3H and 3I, autofluorescence and AGE protein levels increased in aging fibroblasts compared to young fibroblasts, which supported aging fibroblasts, including lipofuscin-like particles in Figure 3A, and treatment with lotus germ extract decreased autofluorescence and AGE protein levels in aging fibroblasts. Therefore, lotus germ extract treatment could restore age-dependent disrupted protein homeostasis in aging cells.

It has been previously reported that aggregated proteins suppressed mitochondrial membrane potential [13] and lipofuscin suppressed autophagy [21]. Our results indicated that disruption of proteostasis induced AGEs and lipofuscin in aging fibroblasts. Therefore, we tried to identify the triggers of the disruption of proteostasis. Heat shock proteins (HSPs) have a central role in protein folding and suppressed protein aggregation [11]. We confirmed HSPs mRNA expression levels in different stages of proliferative and replicative aging fibroblasts. As shown in the Supplementary Figure 3, the expression of HSC70, HSP72, HSP90β, and HSP60 mRNA is suppressed in the middle passage of fibroblasts. This indicates that suppression of HSPs expression in aging process stimulates disruption of proteostasis. Therefore, during the proliferative and replicative aging process, the downregulation of the expression of HSPs disrupts proteostasis. This process leads to an increase in various abnormal agglomerates, such as lipofuscin and aggregating proteins, which are associated with a decreased mitochondrial membrane potential and inhibition of autophagy.

### Lotus germ extract activates the DAPK1-Beclin1 signaling pathway

**Figure 4:**
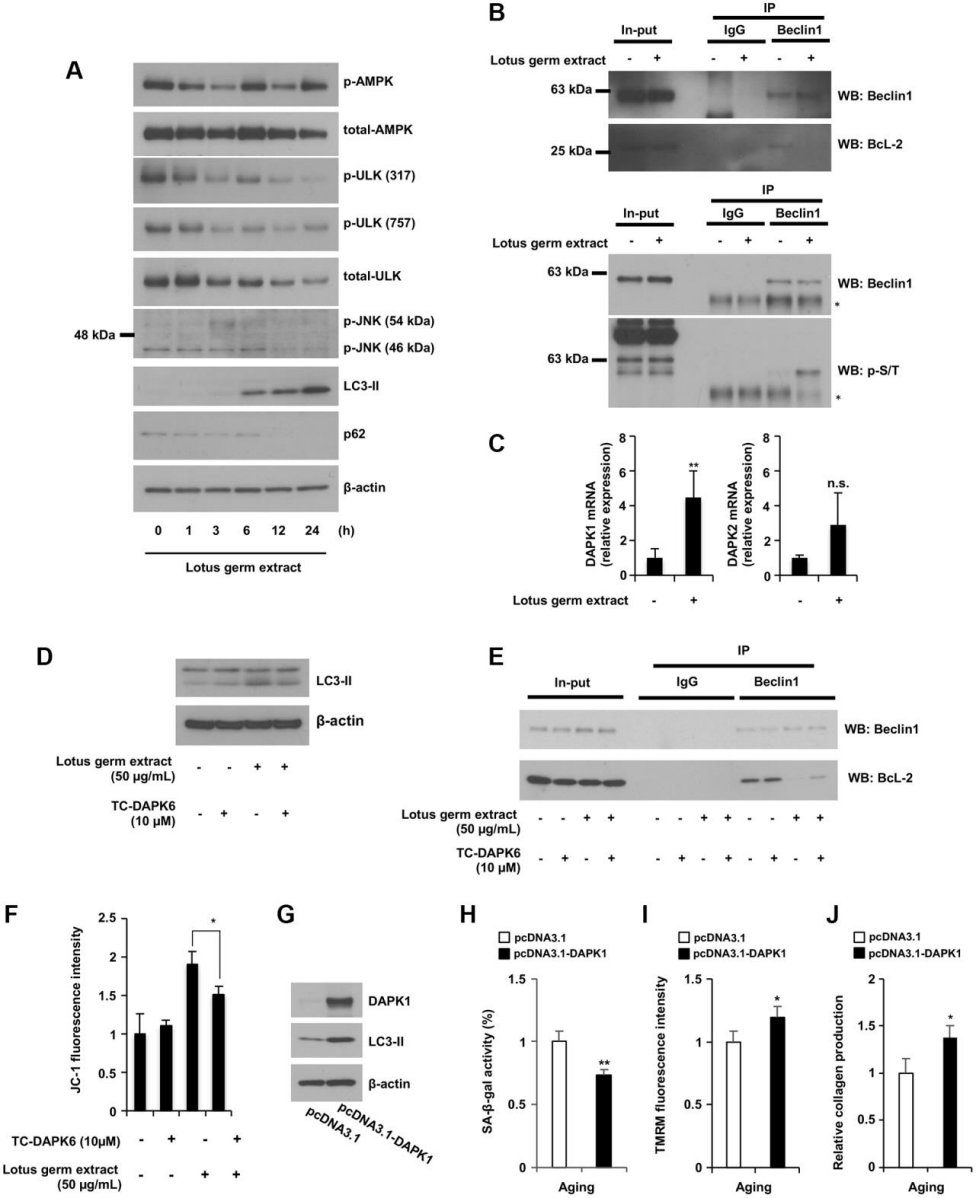
**Lotus germ extract activated the JNK and DAPK1-Beclin1 pathways.** (**A**) Lotus germ extract phosphorylated JNK at an early time point. Aging NB1RGB cells were treated with 50 μg/mL lotus germ extract for the indicated times and subjected to immunoblotting using the indicated antibodies. (**B**) Lotus germ extract inhibited the Beclin1 and BcL-2 interaction via Beclin1 phosphorylation. Aging NB1RGB cells were treated with DMSO (−) or 50 μg/mL lotus germ extract (+) for 24 h, and proteins were crosslinked with DSP prior to protein extraction. A coimmunoprecipitation assay was performed with cell lysates using the indicated antibodies, followed by western blotting. (**C**) Lotus germ extract induced DAPK1 expression. Aging NB1RGB cells were treated with DMSO (−) or 50 μg/mL lotus germ extract (+) for 24 h and subjected to real-time quantitative PCR. (**D**–**F**) DAPK1 plays an important role in the effects of lotus germ extract. Aging NB1RGB cells were treated with DMSO (−) or 10 μM TC-DAPK6 and/or 50 μg/mL lotus germ extract for 24 h and subjected to immunoblotting using the indicated antibodies (**D**), coimmunoprecipitation assay (**E**) or JC-1 activity measurementṣ (**F**). (**G**–**J**) The overexpression of DAPK1 stimulates the mitochondrial activity in aging fibroblast cells. Aging fibroblast cells transfected with pcDNA3.1 or pcDNA3.1-DAPK1 for 48 h were subjected to immunoblotting using the indicated antibodies (**G**), SA-β-gal activity measurement (**H**), TMRM activity measurement (**I**), or collagen production (**J**). Data are presented as the mean ± SD of three simultaneously performed experiments (**C**, **F**, **H**–**J**). Each *P* value was calculated using two-way ANOVA; n.s.: not significant, ^*^P < 0.05, ^**^P < 0.01.

AMPK signaling is a major pathway for the induction of autophagy; specifically, the inactivation of mTORC1 and activation of unc-51-like kinase (ULK) are directly or indirectly regulated by AMPK [22]. Thus, we investigated whether lotus germ extract affects AMPK activity. As shown in Figure 4A, lotus germ extract did not induce the phosphorylation of AMPK (Thr172) or activation of ULK (via Ser317 phosphorylation and Ser757 dephosphorylation), but phosphorylation of JNK, which induces autophagy via the phosphorylation of Beclin1, was slightly increased at 3 h upon treatment with lotus germ extract, after which LC3-II expression increased, p62, a typical selective substrate for autophagy, expression decreased, and lysosome-degradation activity was maintained in the aging fibroblasts.

Beclin1 plays a central role during the autophagy process [22]. Under normal conditions, Bcl-2 binds Beclin1 and inhibits autophagosome formation; however, the phosphorylation of Beclin1 releases it from Bcl-2, and Beclin1 can then carry out its function in autophagy [23]. Therefore, we tested whether lotus germ extract affects the binding of Beclin1 and Bcl-2 and the phosphorylation of Beclin1. Immunoprecipitation showed that Beclin1 did not bind Bcl-2, and Beclin1 was phosphorylated under lotus germ extract treatment (Figure 4B). We confirmed whether the lotus germ extract affects Beclin1 expression level. As shown in Supplementary Figure 2F, the lotus germ extract did not affect the Beclin1 mRNA expression level, and the Beclin1 protein expression level showed almost no change (Figure 4B; shown input lanes). The phosphorylation of JNK was detected at only 3 h; thus, we aimed to identify other factors involved in Beclin1 phosphorylation. Death-associated protein kinase 1 (DAPK1) expression was induced by lotus germ extract, but DAPK2 expression was not (Figure 4C). DAPK1 promotes noncanonical autophagy via the phosphorylation of Beclin1 [24], and we determined whether the induction of DAPK1 expression is involved in lotus germ extract-induced autophagy. Treatment with a DAPK1 inhibitor (TC-DAPK6) suppressed lotus germ extract-induced upregulation of LC3-II expression and disintegration of the Beclin1 and Bcl-2 complex in aging fibroblasts (Figure 4D and 4E). Furthermore, inhibition of DAPK1 activity suppressed the reactivation of mitochondrial function in aging fibroblasts (Figure 4F). We demonstrated the effect of overexpression of DAPK1 for several aging phenotypes. As shown in Figure 4G–4J, the overexpression of DAPK1 induced the LC3-II expression, inhibited SA-β-gal activity, and stimulated mitochondrial ΔΨ*m* and collagen production. These results indicated that the effect of the DAPK1 upregulation expression was similar to the lotus germ extract-induced antiaging effect in aging fibroblasts, suggesting that the induction of DAPK1 is a crucial signal induction for the antiaging effect of the lotus germ extract. The data shown in Figure 4 indicate that the lotus germ extract induces autophagy via the DAPK1-Beclin1 pathway, a noncanonical autophagy pathway.

### Lotus germ extract modulates age-dependent epigenetic changes, which are related to the induction of DAPK1 expression

**Figure 5:**
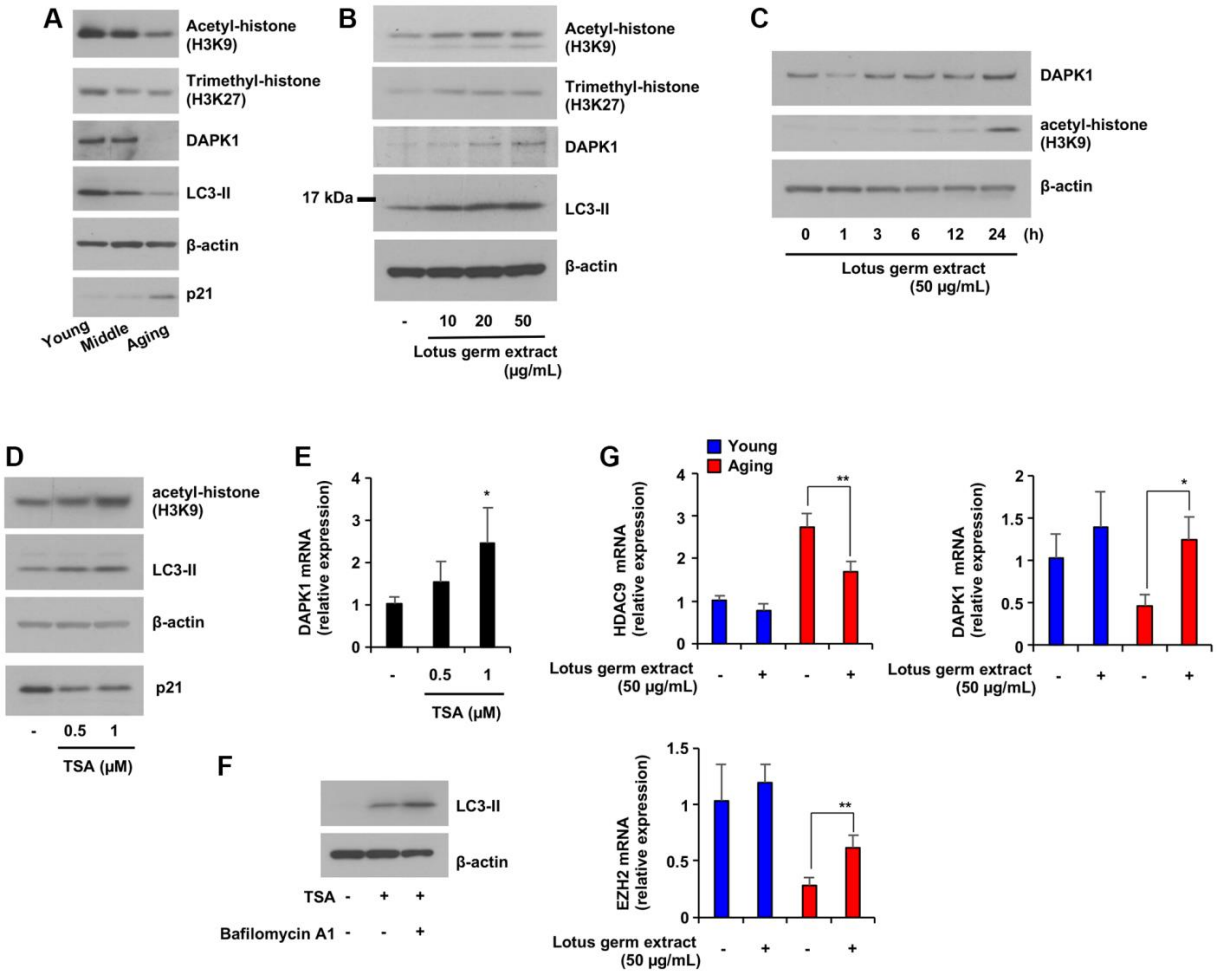
**Lotus germ extract induced DAPK1 expression via the inhibition of age-dependent histone acetylation.** (**A**) Cellular aging suppressed DAPK1 expression and LC3-II expression. NB1RGB cells at several passages were subjected to immunoblot assays using the indicated antibodies. (**B**) Lotus germ extract induced histone acetylation. Aging NB1RGB cells were treated with the indicated concentration of lotus germ extract for 24 h and subjected to immunoblot assays using the indicated antibodies. (**C**) Aging NB1RGB cells were treated with the indicated concentration of lotus germ extract for the indicated time and subjected to immunoblot assays using the indicated antibodies. (**D** and **E**) Inhibition of histone acetylation increased DAPK1 mRNA expression in aging NB1RGB cells. Cells were treated with DMSO (−) or the indicated concentration of TSA for 24 h and subjected to immunoblotting assay (**D**) or q-PCR assay (**E**). (**F**) The treatment with TSA stimulates autophagosome synthesis. Aging NB1RGB cells were treated with DMSO (−) or 1 μM TSA for 24 h, followed by treatment with or without bafilomycin A1 (1 μg/mL) for 2 h. Cells were subjected to immunoblotting using the indicated antibodies. (**G**) Lotus germ extract specifically modulated epigenetic control genes in aging cells. Young and aging NB1RGB cells were treated with DMSO (−) or 50 μg/mL lotus germ extract (+) for 24 h and subjected to qPCR. Data are presented as the mean ± SD of three simultaneously performed experiments (**E**, **G**). Each *P* value was calculated using two-way ANOVA; n.s.: not significant, ^*^P < 0.05, ^**^P < 0.01.

Epigenetic alterations represent one of the crucial mechanisms behind the deteriorated cellular function observed during the aging process. Among the histone modifications known to affect longevity, the most important are acetylation and methylation of lysine residues via HDACs or EZH2 [14, 25]. The total levels of trimethylation of H3K27 (H3K27me) and acetylation of H3K9 (H3K9ac) were decreased in aging HDFs compared to young HDFs; however, contradictory results have been reported, as both suppression and stimulation of acetylation and/or methylation of histones has been shown to lead to longevity [25–28]. On the other hand, the expression of DAPK1 regulates epigenetic alterations, among which histone deacetylation suppresses DAPK1 mRNA expression, and the inhibition of HDAC9, which deacetylates H3K9ac, stimulates DAPK1 expression [29, 30]. Thus, we examined whether lotus germ extract would affect the levels of histone acetylation and methylation and the expression of factors that regulate them, which were restored to that of young fibroblasts in aging fibroblasts. As shown in Figure 5A and Supplementary Figure 1F, the expression of H3K9ac and DAPK1 was decreased, the expression of p21 was increased in aging fibroblasts, and the expression of H3K27me and LC3-II was decreased in a culture time-dependent manner. Furthermore, the results showed that lotus germ extract treatment of aging fibroblasts induced the expression of H3K9ac, H3K27me, DAPK1 and LC3-II in a dose-dependent manner (Figure 5B). In Figure 5B, the LC3-I band is not clear. We demonstrated whether the lotus germ extract induced the upregulation of LC3-II and was converted from LC3-I or increased expression of LC3 mRNA. As shown in Supplementary Figure 2G, lotus germ extract treatment did not change LC3 mRNA expression level, suggesting that most LC3-II were converted from LC3-I. The levels of H3K9ac and DAPK1 protein expression were correlated, as shown in Figure 5A and 5B. We examined the temporal correlation between these factors and found that lotus germ extract strongly induced the acetylation of H3K9 from 12 h to 24 h, and DAPK1 was induced at 24 h, indicating that lotus germ extract increased the acetylation of H3K9 and then induced DAPK1 (Figure 5C and Supplementary Figure 1E). To confirm this result, treatment with a global HDAC inhibitor, trichostatin A (TSA), increased H3K9ac levels, LC3-II expression and DAPK1 mRNA expression and decreased p21 expression in aging fibroblasts (Figure 5D and 5E), suggesting that the acetylation level of H3K9 was regulated by the DAPK1 mRNA expression level. As shown in Figure 5F, the co-treatment with TSA and bafilomycin A1 increased LC3-II protein levels compared to TSA alone, indicating that the TSA treatment induced autophagosome synthesis. To further understand the molecular mechanism by which lotus germ extract induced the acetylation of histones, we focused on the HDAC family, as the deacetylation of some histones is upregulated by cellular aging. As shown in Figure 5G and Supplementary Figure 1G, HDAC9 was upregulated in aging fibroblasts compared to young fibroblasts, and lotus germ extract suppressed HDAC9 expression. Additionally, lotus germ extract restored the expression levels of some HDACs (HDAC1, HDAC8 and HDAC10) and the histone methylation regulatory factor EZH2 in aging fibroblasts to the levels observed in younger fibroblasts (Figures 5F and Supplementary Figure 4). In conclusion, expression of the histone modification factors HDAC9 and EZH2 was changed in aging fibroblasts. Treatment with lotus germ extract increased the expression of these factors to the level observed in the young fibroblasts and induced the expression of DAPK1, which was reduced in aging fibroblasts.

### Lotus germ extract rejuvenated aging fibroblasts

**Figure 6:**
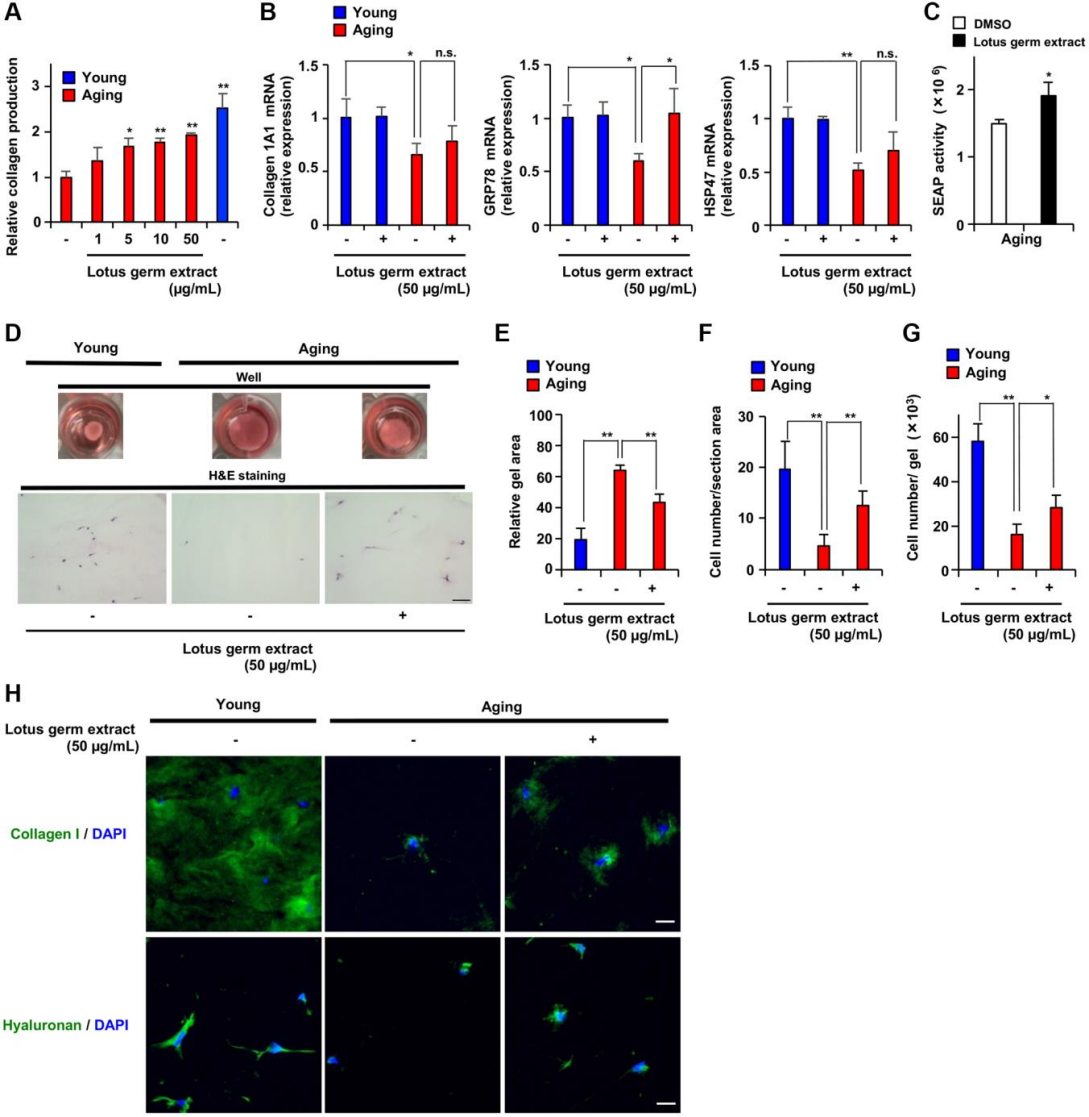
**Lotus germ extract stimulated collagen production and proliferative ability in aging cells in 3-dimensional gel culture.** (**A**) Young NB1RGB cells, or Aging NB1RGB cells were treated with DMSO (−) or the indicated concentration of lotus germ extract for 72 h and subjected to measurement of collagen content. (**B** and **C**) Lotus germ extract stimulated GRP78 expression and protein folding in the ER. (**B**) Young and aging NB1RGB cells were treated with DMSO (−) or 50 μg/mL lotus germ extract (+) for 24 h and subjected to qPCR. (**C**) Aging NB1RGB cells expressing SEAP were transduced with a pSEAP2-Control vector and washed 18 h after transduction, and the cells were treated with DMSO (−) or 50 μg/mL lotus germ extract for 24 h. The medium was then changed, and the cells were cultured for another 18 h. Culture media were analyzed for SEAP activity, and luminescence was normalized to cell number. (**D**–**H**) Lotus germ extract stimulated collagen production and proliferative ability in 3-dimensional atelocollagen gel culture. Aging NB1RGB cells were treated with DMSO (−) or 50 μg/mL lotus germ extract for 72 h. Equal numbers of these cells and young NB1RGB cells were cultured in atelocollagen gel and treated with or with 50 μg/mL lotus germ extract for 10 days. (**D**) Representative image showing collagen gel contraction (upper panel). H&E staining of 3-dimensional atelocollagen gel sections. Scar bar: 100 μm (lower panel). (**E**) Gel surface area. (**F**) The cell density in a 3-dimensional gel was calculated by using H&E staining. (**G**) The cell number in the 3-dimensional gel was counted based on degradation of the atelocollagen gel. (**H**) Extracellular collagen and hyaluronan were measured by immunofluorescence staining. Scar bar: 20 μm. Data are presented as the mean ± SD of three simultaneously performed experiments (**A**–**C**, **E**–**G**). Each *P* value was calculated using two-way ANOVA; n.s.: not significant, ^*^P < 0.05, ^**^P < 0.01.

**Figure 7:**
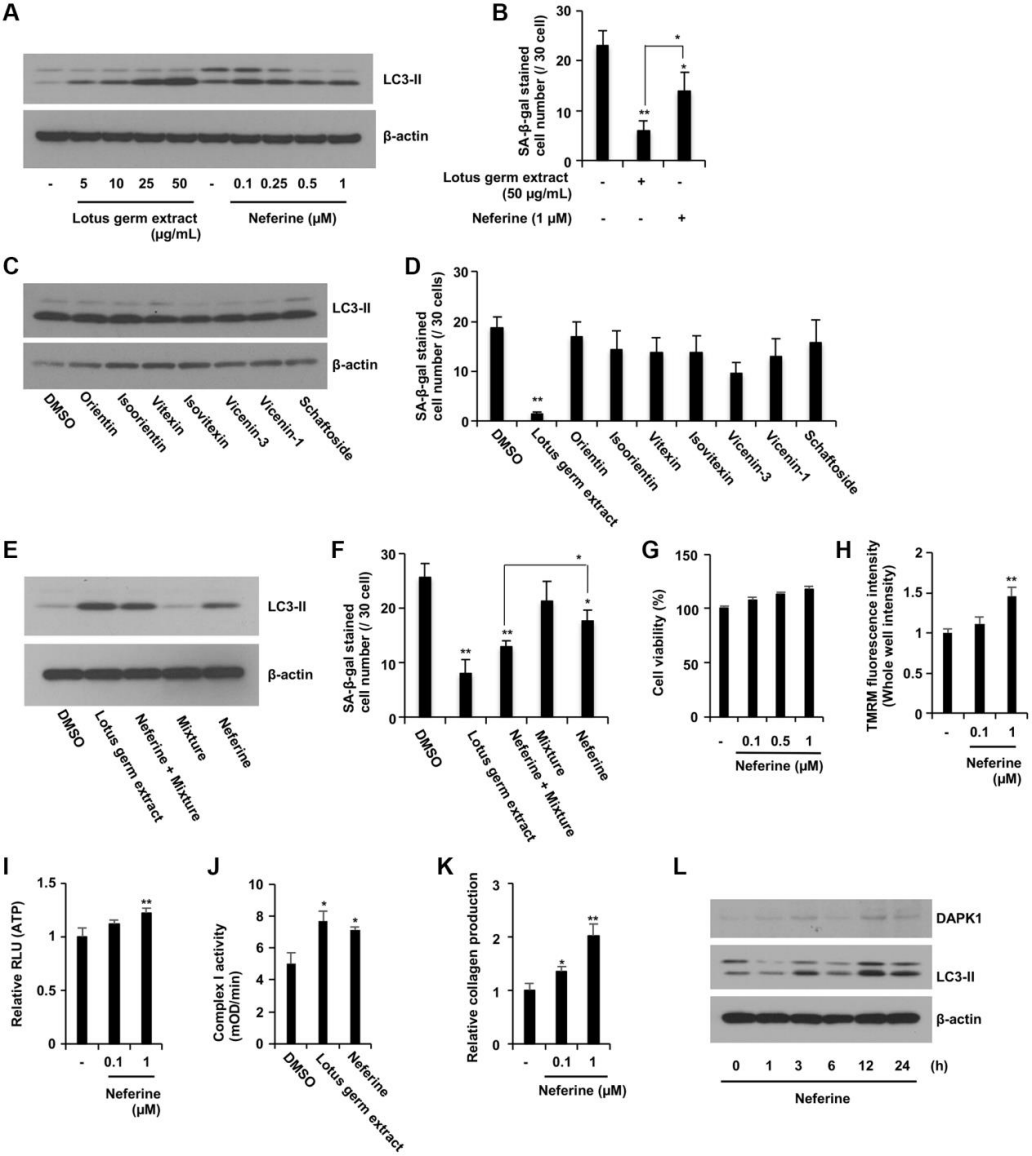
**Neferine and several flavonoids play a central role in the antiaging effects of lotus germ extract.** (**A** and **B**) Neferine treatment induced autophagy and suppressed SA-β-gal activity. Aging NB1RGB cells were treated with DMSO (−) or the indicated concentration of lotus germ extract or neferine for 24 h and subjected to immunoblot (**A**) or SA-β-gal activity (**B**) assays. Lotus germ extract at 50 μg/mL included 1 μM neferine. (**C** and **D**) Several polyphenols included in lotus germ extract did not induce autophagy or suppress SA-β-gal activity. Aging NB1RGB cells were treated with DMSO (-) or the indicated compounds at 10 μM for 24 h and subjected to immunoblot (**C**) or SA-β-gal activity (**D**) assays. The polyphenol mixture (0.11 μM orientin and 0.76 μM vicenin-1) stimulated neferine-dependent induction of autophagy and suppressed SA-β-gal activity. Aging NB1RGB cells were treated with DMSO (−) or 50 μg/mL lotus germ extract or 1 μM neferine with or without a mixture of 0.76 μM vicenin-1 and 0.11 μM orientin for 24 h and subjected to immunoblotting (**E**) or SA-β-gal activity (**F**) assays. (**G**–**J**) Neferine rejuvenates aging fibroblasts. Aging NB1RGB cells were treated with DMSO (−), 50 μg/mL lotus germ extract, neferine at the indicated concentration (**G**–**I**, **K**), or 1 μM (**J**) for 24 h. (**G**) Cell viability was determined using an MTT assay. (**H**) ΔΨ*m* was determined by the TMRM red fluorescence intensity, indicating activated mitochondria. (**I**) ATP levels were determined using a CellTiter-Glo assay. (**J**) Mitochondrial complex I activity was determined. (**K**) Collagen productions were determined. (**L**) Aging NB1RGB cells were treated with 1 μM neferine for the indicated times and subjected to immunoblotting using the indicated antibodies. Data are presented as the mean ± SD of three simultaneous experiments (**B**, **D**, **F**–**K**). Each *P* value was calculated using two-way ANOVA; ^*^P < 0.05, ^**^P < 0.01.

We showed that lotus germ extract restored several age-dependent changes in aging fibroblasts to more closely resemble younger fibroblasts. Thus, we hypothesized that lotus germ extract can rejuvenate the functions of aging fibroblasts. First, we focused on collagen production. As shown in Figure 6A, the production of total collagen protein was significantly decreased in aging fibroblasts compared to young fibroblasts and increased by 5–50 μg/mL lotus germ extract treatment. This effect was suppressed by a DAPK1 inhibitor (Supplementary Figure 5). Figures 2B, 2I, 5B, 6A, and 7A indicated that most aging phenotypes decreased from 10 μg/mL lotus germ extract treatment in aging fibroblasts.

The amount of collagen production regulates both collagen mRNA expression and protein folding capacity in the endoplasmic reticulum (ER), which involves ER chaperones such as GRP78 and HSP47. Treatment with lotus germ extract did not change collagen 1A1 or HSP47 mRNA expression but increased GRP78 mRNA expression in aging fibroblasts, suggesting that ER protein folding capacity might have been increased by lotus germ extract (Figure 6B). We next investigated the effect of lotus germ extract on the secretory output of the ER using the secreted alkaline phosphatase (SEAP) assay [31]. We expressed SEAP in aging fibroblasts and monitored its secretion. Lotus germ extract significantly enhanced the secretion of SEAP (Figure 6C), suggesting that the stimulation of collagen production depends on enhanced secretory function of the ER and was enhanced by lotus germ extract.

Next, we performed a 3-dimensional culture assay using collagen gel to evaluate the functions of aging fibroblasts (proliferation, reorganization and the contraction of extracellular matrix) in an environment that more closely simulated *in vivo* conditions. As shown in Figure 6D–6G and Supplementary Figure 6, young fibroblasts contracted collagen gel and proliferated in 3-dimensional culture, but aging fibroblasts showed decreased contraction activity and proliferation, and the cell number in 3-dimensional culture was lower for aging fibroblasts than for younger fibroblasts. Lotus germ extract treatment promoted these activities in aging fibroblasts (Figure 6D–6G and Supplementary Figure 6). An immunofluorescence staining assay revealed that collagen and hyaluronan, a factor in the extracellular matrix, were increased in 3-dimensional culture upon treatment with lotus germ extract (Figure 6H), indicating that lotus germ extract restored extracellular matrix-remodeling activity in aging fibroblasts. Altogether, the data shown in Figure 6 indicate that lotus germ extract treatment rejuvenated aging fibroblasts by restoring the various phenotypes of aging via activation of DAPK1-Beclin1 signaling.

### Neferine is one of the potential active molecule(s) in the lotus germ extract

To identify the active molecule(s) in the lotus germ extract, we tested several flavonoid and alkaloid compounds, such as neferine, which is known to be included in lotus germ extract [32, 33]. We confirmed that the neferine content in the lotus germ extract that we used was 1.19% and that almost 1 μM neferine was contained in 50 μg/mL lotus germ extract. We compared the ability of these compounds and lotus germ extract to induce autophagy and suppress the number of SA-β-gal positive cell; neferine induced autophagy and decreased the number of SA-β-gal-positive cells, but these effects were less pronounced than those of lotus germ extract, and none of the flavonoids had an effect on these parameters (Figure 7A–7D). In addition, we determined the flavonoid content in lotus germ extract. HPLC analysis revealed that 50 μg/mL lotus germ extract included 0.76 μM vicenin-1, 0.11 μM orientin, 0.04 μM isoorientin, 0.02 μM vitexin and 0.02 μM isovitexin, but scaftoside and vicenin-3 could not be separated. Interestingly, the combination of 1 μM neferine, 0.11 μM orientin and 0.76 μM vicenin-1 (mixture: 0.11 μM orientin and 0.76 μM vicenin-1) had almost the same effect as lotus germ extract on the induction of autophagy and decreased the number of SA-β-gal-positive cells in aging cells (Figure 7E and 7F).

Next, we demonstrated whether the neferine, a potential active ingredient, affects critical parameters involved in aging fibroblasts. In Figure 7G and 7H, neferine increased the ΔΨ*m* in aging fibroblasts, which was dose-dependent without inducing cell death. As shown in Figure 7I–7K, the neferine treatment significantly increased ATP production, collagen production, and mitochondrial complex I activity in aging fibroblasts. Furthermore, the results showed that neferine treatment of aging fibroblasts induced the expression of DAPK1 and LC3-II in 12–24 h (Figure 7L). Therefore, neferine is mainly responsible for the effects of the lotus germ extract, but several flavonoids synergistically stimulate the effects of neferine. We showed the full Western Blot images in the Supplementary Figure 7.

## DISCUSSION

**Figure 8:**
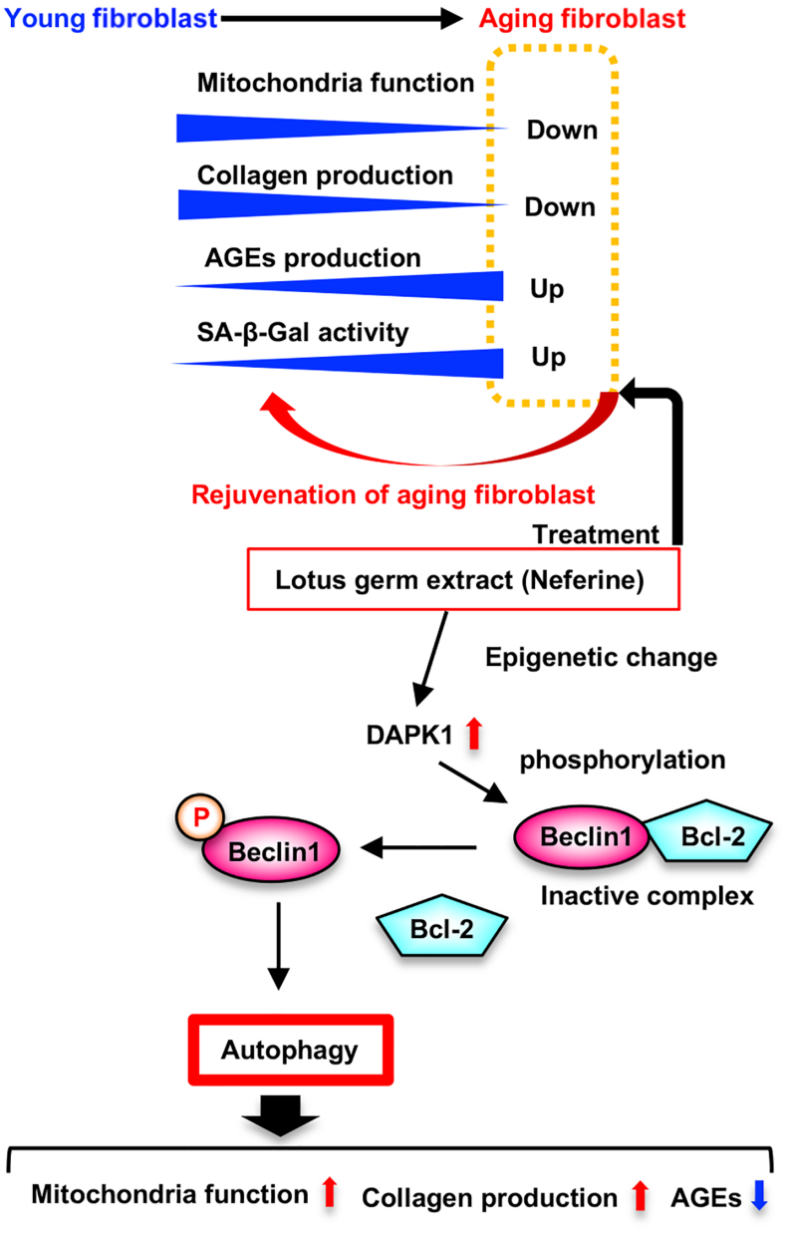
**Model for rejuvenated effect of lotus germ extract (Neferine) in aging fibroblast.** Aging fibroblasts downregulates mitochondria function and collagen production, and upregulates AGEs production and SA-β-gal activity. Lotus germ extract (Neferine) treatment rejuvenated aging fibroblasts by restoring the various phenotypes of aging via activation of DAPK1-Beclin1 signaling induced autophagy.

Here, we have identified lotus germ extract as a new anti-aging material containing several active compounds with coordinated effects. We discovered that mitochondrial dysfunction occurred earlier than the induction of DNA damage signaling, and reactivation of mitochondrial function rejuvenated aging fibroblasts, which improved collagen-production capacity and contractile ability, which were reduced over time via the restoration of epigenetic changes and degradation of lipofuscin-like aggregation bodies, including AGEs. Through a pharmacological approach, we identified the DAPK1-Beclin1 pathway as an important autophagy induction signaling pathway in aging HDFs, and the induction of autophagy plays a crucial role in the antiaging effects of lotus germ extract (Figure 8).

Numerous studies have suggested that mitochondrial dysfunction stimulates cellular aging, suggesting the involvement of mtDNA mutations, disruption of proteostasis and reduced mitochondrial biogenesis as possible mechanisms, but contradictory reports on the related mechanisms are available, and the issue remains unresolved [7, 34]. In this study, we focused on the disruption of proteostasis in aging fibroblasts by electron microscopy analysis, and ELISA revealed that lipofuscin-like aggregates and AGEs were increased in fibroblasts in the early stage of aging. Li et al. reported that proteostatic stress induces mitochondrial dysfunction and cellular aging, leading to the direct accumulation of abnormal proteins in mitochondria [13]. The accumulation of lipofuscin in the cytosol also indicates increased unfolded protein levels via disruption of the proteostatic system, and lipofuscin is a strong target for autophagy; thus, attacking lipofuscin is ineffectual for lysosomal enzymes, and the number of defective mitochondria increases because the degradation of damaged mitochondria via autophagy has been impeded [21]. We showed that lotus germ extract degraded accumulated lipofuscin and AGEs, which inhibit mitochondrial function in aging fibroblasts. Together, the findings show that the lotus germ extract restored aging-dependent mitochondrial dysfunction by restoring proteostasis via the induction of extensive autophagy. These effects are significant considering the potential antiaging target of the lotus germ extract.

Autophagy is an important biological system that maintains proteostasis by degrading abnormal proteins and dysfunctional organelles [22]. Autophagic disability is involved in stimulation of the aging process, and autophagy is known to decrease with increasing age [35]. ATG5, the essential autophagy induction gene, transgenic mice had antiaging phenotypes and a lifespan extension of about 20% [36]. Several well-known antiaging compounds, such as rapamycin, metformin, resveratrol, and spermidine, have been proven to activate autophagy directly [11]. We observed the downregulation of DAPK1 expression and a reduction in autophagy, which might relate to a decreased capacity to degrade abnormal substances in aging fibroblasts. Furthermore, the overexpression of DAPK1 induced autophagy and restored mitochondria function in this study. DAPK1 induces a type of noncanonical autophagy, which is induced by the upregulation of DAPK1 expression induced via viral infection [24], and DAPK1 directly phosphorylates Beclin1, thereby releasing it from Bcl-2 [23]. Thus, the lotus germ extract-induced activation of the DAPK1-Beclin1 pathway, which is the primary signal activating autophagy and is a potential target for the antiaging effect of the extract in aging fibroblasts. The expression of DAPK1 is known to be regulated by epigenetic changes; DAPK1 expression is induced by increased H3K9ac, and the activation of HDACs suppresses DAPK1 expression [29]. However, the DAPK1 expression level in aging fibroblasts has not been determined. We show that DAPK1 expression was suppressed over time by the aging process, which decreased H3K9ac levels, and the HDAC inhibitor and lotus germ extract restored DAPK1 expression by upregulating H3K9ac levels in aging fibroblasts, indicating that the upregulation of DAPK1 might specifically induce autophagy in aging cells. HDAC9 expression levels were upregulated, and H3K9ac levels were downregulated in aging cells, and these changes impaired autophagy [26]. Lotus germ extract suppressed aging-dependent upregulation of HDAC9 expression, which might be related to increased H3K9ac levels. However, additional experiments are needed to identify the molecular mechanism by which lotus germ extract induces the acetylation of H3K9.

The collagen-production process is transcribed, translated, and then specific structures are formed in the ER and secreted out of the cell. One of the critical factors in collagen production is the formation of particular structures in the ER, which suppresses collagen production when its function is impaired [36]. It has also been reported that impaired mitochondrial function downregulates collagen production, enhances degradation, and induction of autophagy stimulates collagen production by degrading unfolded and aggregated proteins [37]. The results of this study suggest that the lotus germ extract might stimulate collagen production by degrading lipofuscin included in unfolded and aggregated proteins and activating mitochondrial function. The DAPK1-Beclin1 pathway-induced autophagy plays a central role in these processes. The lotus germ extract also stimulated collagen production by upregulating the expression of ER chaperones and enhancing the protein folding capacity in ER. Nonetheless, this molecular mechanism still needs to be elucidated. There has been less analysis on how epigenetic changes are involved in collagen production. In this study, the lotus germ extract treatment induced epigenetic changes. Still, collagen 1A1 mRNA expression level did not affect it, suggesting that epigenetic changes regulate collagen production through indirect pathways, such as the upregulation of DAPK1 expression.

Many studies to determine the pharmacological effects of lotus germ extract have been conducted, and its anti-obesity, anticancer and anti-inflammatory effects have been identified [15]. Lotus germ extract has therapeutic effects on Alzheimer’s disease, an aging-related disease, but its effects on time-dependent organismal and cellular aging have not been clarified. This study indicates that several flavonoids stimulate the neferine effect. Nonetheless, neferine is a potential active ingredient in lotus germ extract that exerts antiaging effects. We expect that the direct target of the antiaging effect of lotus germ extract will be identified by investigating the antiaging effect of neferine using pharmacological and chemical biological methods.

Overall, these findings establish the rejuvenating effect of lotus germ extract on aging fibroblasts via the induction of autophagy to clear abnormal proteins and agglutinates. Moreover, DAPK1-Beclin1 signaling-induced autophagy might be a specific target for the antiaging effect of lotus germ extract in aging fibroblasts.

## MATERIALS AND METHODS

### Cell lines and generation of stable cell lines

NB1RGB (Riken BRC) and IMR90 (JCRB Cell Bank) cells were maintained in MEMα supplemented with 10% FBS, 100 U/mL penicillin, and 100 μg/mL streptomycin. All cell cultures were passaged (1:4 to 3) and maintained at 37°C with 5% CO_2_. We defined three types of each cell line according to the number of days in culture: NB1RGB: young cells: from 8 to 20 days, middle passage: from 30 to 40 days, and aging cells: from 60 to 70 days; IMR90: young cells: from 6 to 12 days, middle passage: from 18 to 25 days, and aging cells: from 30 to 40 days.

NB1RGB cells were transiently transfected using lipofection with plasmids expressing DAPK1 [pcDNA3.1-DAPK1, cloned from pDONR223-DAPK1, which was a gift from William Hahn and David Root (Addgene plasmid # 23480; http://n2t.net/addgene:23480; RRID: Addgene_23480)] using primers (5′-ATGACCGTGTTCAGGCAGGAAAACG-3′ and 5′-TCACCGGGATACAACAGAGCTAATGGAATT-3′).

### Extract preparation

Lotus (*N. nucifera* Gaertn.) germs (200 g) were extracted with 50% ethanol under reflux for 2 h. The mixture was filtered through diatomite. The filtrate was concentrated under reduced pressure at 60°C. The residue was freeze-dried, and 23.1 g of the lotus germ extract was obtained (yield 11.6%).

### Real-time quantitative PCR

Real-time quantitative PCR (qRT–PCR) was performed as previously described [38]. Total RNA was normalized in each reaction using *β-actin* cDNA as an internal standard. The primers used are listed in Table 2.

**Table 2:** List of primer for real time q-PCR.

*p21*	*5′-TGGACCTGTCACTGTCTTGT-3′*	*5′-GGCGTTTGGAGTGGTAGAAA-3′*
*p16*	*5′-CAGGTCATGATGATGGGCAG-3′*	*5′-ACCAGCGTGTCCAGGAAG-3′*
*PLK-1*	*5′-CACAGTGTCAATGCCTCCAA-3′*	*5′-TTGCTGACCCAGAAGATGG-3′*
*CENP-F*	*5′-GTAGAGGACCAACACCTGCTACC-3′*	*5′-GTCAGCAAACCCTTTCTTTACAACT-3′*
*IL-6*	*5′-CCAGCTATGAACTCCTTCTC-3′*	*5′-GCTTGTTCCTCACATCTCTC-3′*
*TNF-α*	*5′-AGCCTCTCTTCTCCTTCCTGATCGT-3′*	*5′-GGCTGATTAGAGAGAGGTCCCTG-3′*
*EZH2*	*5′-GCAACACCCAACACTTATAAGC-3′*	*5′-CTCCCTCCAAATGCTGGTA-3′*
*MYC*	*5′-GGATTCTCTGCTCTCCTCGAC-3′*	*5′-TTGTTCCTCCTCAGAGTCGC-3′*
*DAPK1*	*5′-ATGATCCCACGTCAATCCAT-3′*	*5′-ACCGAAGGCTATGGGTTCTT-3′*
*DAPK2*	*5′-TCCTGGATGGGGTGAACTAC-3′*	*5′-CAGCTTGATGTGTGGAATGG-3′*
*GRP78*	*5′-TAGCGTATGGTGCTGCTGTC-3′*	*5′-TTTGTCAGGGGTCTTTCACC-3′*
*COL1A1*	*5′-CAAGAGGCATGTCTGGTTCG-3′*	*5′-TAGGTGATGTTCTGGGAGGC-3′*
*HSP47*	*5′-GACCACCCCTTCATCTTCCT-3′*	*5′-ACTCGTCTCGCATCTTGTCA-3′*
*β-actin*	*5′-GGACTTCGAGCAAGAGATGG-3′*	*5′-AGCACTGTGTTGGCGTACAG-3′*
*HDAC1*	*5′-AACAGGCCATCGAATACTGG-3′*	*5′-GGAAATCTATCGCCCTCACA-3′*
*HDAC2*	*5′-TAAATCCAAGGACAACAGTGG-3′*	*5′-GGTGAGACTGTCAAATTCAGG-3′*
*HDAC3*	*5′-TAGACAAGGACTGAGATTGCC-3′*	*5′-GTGTTAGGGAGCCAGAGCC-3′*
*HDAC4*	*5′-GGTTTATTCTGATTGAGAACTGG-3′*	*5′-ATTGTAAACCACAGTGCTCGC-3′*
*HDAC5*	*5′-CAGCAGGCGTTCTACAATGA-3′*	*5′-GTCCTCCACCAACCTCTTCA-3′*
*HDAC6*	*5′-CGAGCTGATCCAAACTCCTC-3′*	*5′-GGTCCTGAGACTCCCAATCA-3′*
*HDAC7*	*5′-CCCAGCAAACCTTCTACCAA-3′*	*5′-AAGCAGCCAGGTACTCAGGA-3′*
*HDAC8*	*5′-GGTGACGTGTCTGATGTTGG-3′*	*5′-AGCTCCCAGCTGTAAGACCA-3′*
*HDAC9*	*5′-GCTCAGCTGGTCATTCAACA-3′*	*5′-ACTGCCTGGTTGCTTCAGTT-3′*
*HDAC10*	*5′-GCCGGATATCACATTGGTTC-3′*	*5′-GGCTGGAGTGGCTGCTATAC-3′*
*HSC70*	*5′-TCCCTTGGTATTGAAACTGC-3′*	*5′-AACTTGCCAAGCAGGTTGTT-3′*
*HSP72*	*5′-TGTCGCTGGGGCTGGAGA-3′*	*5′-GCCCCAACAGATTGTTGTCT-3′*
*HSP27*	*5′-CTGACGGTCAAGACCAAGGATG-3′*	*5′-GTGTATTTCCGCGTGAAGCACC-3′*
*HSP90α*	*5′-AGGTGGACTGTTTCCTCTCTC-3′*	*5′-TTGCTCACTTGCTTGCTTGTTG-3′*
*HSP90β*	*5′-GAGATCAAAGACTACAGTCCCT-3′*	*5′-GTTCGTGCTCATACTTGGTC-3′*
*HSP60*	*5′-CGCTGAAGATGTTGATGGAG-3′*	*5′-TTTCTATTGTCACCAAACCCTG-3′*

### Detection of SA-β-gal activity

Cellular SA-β-gal activity was determined using a senescence detection kit (Abcam, Cambridge, England) or 96-well cellular senescence assay (Cell Biolabs, San Diego, CA, USA) according to the manufacturer’s protocols. All images were obtained under a microscope (IX73, Olympus, Tokyo, Japan) and processed using Adobe Photoshop software. Fluorescence was measured on a fluorescence microplate reader (Infinite M200, Tecan, Tokyo, Japan) with 360 nm/485 nm filter pairs. Phenylethyl β-d-thiogalactoside (PETG) is a SA-β-gal inhibitor [39].

### JC-1 or TMRM staining

Mitochondrial membrane potential was assessed using JC-1 (Dojindo, Tokyo, Japan) or TMRM (ThermoFisher, Hampton, NH, USA) staining according to the manufacturer’s protocols and as described in previous reports. All images were obtained with a fluorescence microscope (Olympus, Tokyo, Japan) and processed using the Adobe Photoshop software [40]. Fluorescence was measured on a fluorescence microplate reader (Infinite M200, Tecan, Tokyo, Japan) with 535 nm/590 nm and 485 nm/535 nm filter pairs (JC-1) or 535 nm/590 nm filter pairs (TMRM). The results for JC-1 are shown as the ratio of fluorescence measured at 535 nm/590 nm to that measured at 485 nm/535 nm (aggregate fluorescence to monomer fluorescence).

### Cell viability assay

Cell viability was determined using the 3-(4,5-di-methylthiazol-2-yl)-2,5-diphenyltetrazolium bromide (MTT) method. Cells were administered the indicated treatments and incubated with MTT solution (1 mg/mL) for 2 h. Isopropanol and HCl were then added to final concentrations of 50% and 20 mM, respectively, and the absorbance at 570 nm was measured using a spectrophotometer.

### Cell fluorescence staining

Cells were cultured on glass-bottom culture dishes with or without MitoTracker for 30 min and fixed in 4% formaldehyde. All images were obtained under a microscope (BZ-X800, KEYENCE, Osaka, Japan) and processed using Adobe Photoshop software. The mitochondrial morphology was determined by measuring their aspect ratio using ImageJ [41].

### ROS measurements

Cells were cultured on black-bottom culture plates, washed with phosphate-buffered saline (PBS) to remove the medium and subsequently incubated for 10 min at 37°C in 5 mM MitoSOX Red [42]. After incubation, the cells were washed with PBS. The fluorescence was measured with a microplate reader with a 535 nm/590 nm filter pair.

### ATP assay

Intracellular ATP levels were measured using the CellTiter-Glo 2.0 assay kit (Promega, Madison, WI, USA) following the manufacturers’ instructions.

### Mitochondrial respiratory complex I activity

The Abcam mitochondrial respiratory complex I activity assay (ab109721, Cambridge, MA, USA) was used to perform immunocapture of complex I and measure colorimetric reaction activity. This was made through absorbance increases at 450 nm, according to the manufacturer’s protocol.

### Collagen assay

Cellular collagen levels were measured using a collagen quantitation kit (Cosmo Bio, Tokyo, Japan) following the manufacturers’ instructions. Fluorescence was measured on a fluorescence microplate reader (Infinite M200, Tecan, Tokyo, Japan) with a 360 nm/485 nm filter pair.

### TEM

Cultured cells were fixed with 1% glutaraldehyde in PBS at pH 7.3 for 1 h at 4°C and then postfixed with 1% osmium tetroxide in 0.1 M phosphate buffer at pH 7.3 for 1 h at 4°C, followed by dehydration in a graded series of ethanol. Following dehydration, they were embedded in Epon 812 (TAAB Laboratories Equipment, Berkshire, England) and then observed under a JEM-1400 Plus electron microscope (JEOL, Japan).

### siRNA-mediated gene targeting

NB1RGB cells were transfected with siRNA specific for siATG7 (the siRNA SMARTpool for human ATG7 from Dharmacon, Lafayette, CO, USA) and controls (Santa Cruz, Santa Cruz, CA, USA) using Lipofectamine RNAiMAX transfection reagent (Invitrogen, Carlsbad, CA, USA) according to the manufacturer’s instructions.

### Immunoblotting analysis

Immunoblotting experiments were conducted as previously described [38]. The antibodies used for immunoblotting were specific for the following proteins: LC3b, ATG7, P-AMPK, AMPK (total), P-AKT, AKT (total), P-ULK (Ser757), P-ULK (Ser317), ULK (total), p62, Beclin 1, p-S/T, DAPK1, trimethylhistone (H3K27), acetylhistone (H3K9), and BcL-2 (Cell Signaling); p-JNK (Calbiochem); and β-actin (Sigma). The antibodies were diluted 1:1000, except for anti-β-actin (1:10000). Secondary antibodies were purchased from Promega (anti-rabbit and anti-mouse at 1:5000).

### AGE detection by ELISA

AGE levels were measured using an OxiSelect AGE Competitive ELISA kit (Cell Biolabs, San Diego, CA, USA) following the manufacturer’s instructions. The concentrations and enzyme activities were standardized to the total protein levels.

### Mitophagy assay

To analyze mitophagy, a mitophagy detection kit (Dojindo Molecular Technologies, Rockville, MD, USA) was used according to the manufacturer’s protocol. This kit is composed of Mtphagy Dye, a reagent for the detection of mitophagy. All images were obtained with a fluorescence microscope (Olympus, Tokyo, Japan) and processed using the Adobe Photoshop software. Fluorescence was measured on a fluorescence microplate reader (Infinite M200, Tecan, Tokyo, Japan) with a 535 nm/590 nm filter pair.

### Immunoprecipitation

Immunoprecipitation assays were conducted as previously described [43]. Cells were washed and incubated with PBS containing 1 mM dithiobis [succinimidyl propionate] (DSP) for 30 min, and the reaction was quenched by the addition of 50 mM Tris (pH 8.0) for 3 min. Cells were lysed in Tris lysis buffer, and cellular debris was pelleted by centrifugation at 14,000 rpm for 10 min at 4°C. The primary antibody was covalently immobilized onto protein A/G PLUS-agarose, followed by immunoprecipitation using a crosslink immunoprecipitation kit (Pierce/Thermo Fisher Scientific, Hampton, NH, USA) according to the manufacturer’s protocol. Immunoprecipitated products were incubated with LDS sample buffer containing 50 mM DTT at 95°C for 10 min.

### SEAP assay

Cells were transduced with the pSEAP2-Control vector (Takara Bio, Tokyo, Japan). The cells were washed 24 h after transfection, transferred to plates containing fresh medium, and then cultured for 2 h. Culture supernatants were harvested and assayed for SEAP activity using the Great EscAPe SEAP Reporter System (Takara Bio, Tokyo, Japan).

### 3-Dimensional collagen gel culture

Cells were suspended in type I atelocollagen gel medium (KOKEN, Tokyo, Japan), which was immediately warmed to 37°C to form a gel. After 24 h of incubation, the gels were detached from the culture plate, overlaid with a double volume of DMEM and incubated. Each gel area (pix) was measured using ImageJ (NIH, USA). Atelocollagen gels were digested by 0.2% collagenase and 0.1% trypsin treatment, and then the cells were counted.

### Histological and immunohistochemical analyses

Atelocollagen gels were fixed in 4% buffered paraformaldehyde, embedded in O.C.T. compound, and cryosectioned. For histological examination [hematoxylin and eosin (H&E) staining], the sections were stained first with Mayer’s hematoxylin and then with a 1% eosin alcohol solution. The sections were blocked with 5% goat serum for 15 minutes and incubated for 12 h with anti-collagen I primary antibody (Novotec, JZ Reuver, Netherlands) or biotinylated-hyaluronan-binding protein (HRBP) (Hokudo Co., Ltd., Hokkaido, Japan) in the presence of 5% bovine serum albumin and then incubated for 1 h with FITC-conjugated rabbit IgG (Molecular Probes, Hampton, NH, USA) or FITC-conjugated avidin (Vector Laboratories, Burlingame, CA, USA). Nuclei were stained with DAPI (Roche, Indianapolis, IN, USA). The samples were mounted with Permount (Wako, Tokyo, Japan) or Fluoromount (Sigma) and inspected with the aid of a microscope (BZ-X800, Keyence, Osaka, Japan).

### HPLC analysis

An HPLC system from Shimadzu Corp. (Kyoto, Japan) with an SCL-40 system controller, two LC-40D pumps, a DGU-403 degasser, a SIL-40C autosampler, a CTO-40C column oven and an SPD-M40 PDA detector was used. HPLC separation was achieved on a 150 mm × 4.6 mm i.d., 3 μm YMC-Pack Pro C18 column (YMC, Kyoto, Japan). The mobile phases consisted of (A) water with 0.1% (v/v) trifluoroacetic acid and (B) methanol. The separation was carried out at 40°C under the following conditions: 0–45 min, 20%–50% B, 45–55 min, 50% B, followed by 5-min re-equilibration of the column. The detection wavelength was 282 nm, and the flow rate was 1.0 mL/min. The injection volume was 20 μL. Neferine was identified and quantified with the help of a standard. The neferine standard was dissolved with the initial mobile phase to prepare solutions at 7.84, 39.2 and 196 μg/mL. LGE was dissolved in the initial mobile phase to make a 1.99 mg/mL solution. All solutions were filtered through a PTFE membrane filter (0.45 μm) before injection.

### Statistical analyses

Differences in mean values were evaluated using two-way ANOVA, followed by Tukey’s test, with *P* < 0.05 used to indicate statistical significance.

### Data and materials availability

Additional data related to this paper may be requested from the author.

## Supplementary Materials

Supplementary Figures

## References

[1] López-Otín C, Blasco MA, Partridge L, Serrano M, Kroemer G. The hallmarks of aging. Cell. 2013; 153:1194–217. 10.1016/j.cell.2013.05.039 23746838 PMC3836174

[2] Campisi J, Kapahi P, Lithgow GJ, Melov S, Newman JC, Verdin E. From discoveries in ageing research to therapeutics for healthy ageing. Nature. 2019; 571:183–92. 10.1038/s41586-019-1365-2 31292558 PMC7205183

[3] Hernandez-Segura A, Nehme J, Demaria M. Hallmarks of Cellular Senescence. Trends Cell Biol. 2018; 28:436–53. 10.1016/j.tcb.2018.02.001 29477613

[4] Johmura Y, Yamanaka T, Omori S, Wang TW, Sugiura Y, Matsumoto M, Suzuki N, Kumamoto S, Yamaguchi K, Hatakeyama S, Takami T, Yamaguchi R, Shimizu E, et al. Senolysis by glutaminolysis inhibition ameliorates various age-associated disorders. Science. 2021; 371:265–70. 10.1126/science.abb5916 33446552

[5] Baker DJ, Childs BG, Durik M, Wijers ME, Sieben CJ, Zhong J, Saltness RA, Jeganathan KB, Verzosa GC, Pezeshki A, Khazaie K, Miller JD, van Deursen JM. Naturally occurring p16(Ink4a)-positive cells shorten healthy lifespan. Nature. 2016; 530:184–9. 10.1038/nature16932 26840489 PMC4845101

[6] Gray DA, Woulfe J. Lipofuscin and aging: a matter of toxic waste. Sci Aging Knowledge Environ. 2005; 2005:re1. 10.1126/sageke.2005.5.re1 15689603

[7] Kauppila TES, Kauppila JHK, Larsson NG. Mammalian Mitochondria and Aging: An Update. Cell Metab. 2017; 25:57–71. 10.1016/j.cmet.2016.09.017 28094012

[8] Bratic A, Larsson NG. The role of mitochondria in aging. J Clin Invest. 2013; 123:951–7. 10.1172/JCI64125 23454757 PMC3582127

[9] Zimmermann A, Madreiter-Sokolowski C, Stryeck S, Abdellatif M. Targeting the Mitochondria-Proteostasis Axis to Delay Aging. Front Cell Dev Biol. 2021; 9:656201. 10.3389/fcell.2021.656201 33777963 PMC7991595

[10] Patel SH, Yue F, Saw SK, Foguth R, Cannon JR, Shannahan JH, Kuang S, Sabbaghi A, Carroll CC. Advanced Glycation End-Products Suppress Mitochondrial Function and Proliferative Capacity of Achilles Tendon-Derived Fibroblasts. Sci Rep. 2019; 9:12614. 10.1038/s41598-019-49062-8 31471548 PMC6717202

[11] Kaushik S, Cuervo AM. Proteostasis and aging. Nat Med. 2015; 21:1406–15. 10.1038/nm.4001 26646497

[12] Madeo F, Zimmermann A, Maiuri MC, Kroemer G. Essential role for autophagy in life span extension. J Clin Invest. 2015; 125:85–93. 10.1172/JCI73946 25654554 PMC4382258

[13] Li Y, Xue Y, Xu X, Wang G, Liu Y, Wu H, Li W, Wang Y, Chen Z, Zhang W, Zhu Y, Ji W, Xu T, et al. A mitochondrial FUNDC1/HSC70 interaction organizes the proteostatic stress response at the risk of cell morbidity. EMBO J. 2019; 38:e98786. 10.15252/embj.201798786 30591555 PMC6356068

[14] Ito T, Teo YV, Evans SA, Neretti N, Sedivy JM. Regulation of Cellular Senescence by Polycomb Chromatin Modifiers through Distinct DNA Damage- and Histone Methylation-Dependent Pathways. Cell Rep. 2018; 22:3480–92. 10.1016/j.celrep.2018.03.002 29590617 PMC5915310

[15] Showkat QA, Rather JA, Jabeen A, Dar BN, Makroo HA, Majid D. Bioactive components, physicochemical and starch characteristics of different parts of lotus (*Nelumbo nucifera* Gaertn.) plant: a review. Int J Food Sci Technol. 2021; 56:2205–14. 10.1111/ijfs.14863

[16] Westermann B. Bioenergetic role of mitochondrial fusion and fission. Biochim Biophys Acta. 2012; 1817:1833–8. 10.1016/j.bbabio.2012.02.033 22409868

[17] Klionsky DJ, Abdel-Aziz AK, Abdelfatah S, Abdellatif M, Abdoli A, Abel S, Abeliovich H, Abildgaard MH, Abudu YP, Acevedo-Arozena A, Adamopoulos IE, Adeli K, Adolph TE, et al. Guidelines for the use and interpretation of assays for monitoring autophagy (4th edition)^1^. Autophagy. 2021; 17:1–382. 10.1080/15548627.2020.1797280 33634751 PMC7996087

[18] Perrotta I. Occurrence and characterization of lipofuscin and ceroid in human atherosclerotic plaque. Ultrastruct Pathol. 2018; 42:477–88. 10.1080/01913123.2018.1544953 30465462

[19] Rubinsztein DC, Mariño G, Kroemer G. Autophagy and aging. Cell. 2011; 146:682–95. 10.1016/j.cell.2011.07.030 21884931

[20] Moreno-García A, Kun A, Calero O, Medina M, Calero M. An Overview of the Role of Lipofuscin in Age-Related Neurodegeneration. Front Neurosci. 2018; 12:464. 10.3389/fnins.2018.00464 30026686 PMC6041410

[21] Höhn A, Grune T. Lipofuscin: formation, effects and role of macroautophagy. Redox Biol. 2013; 1:140–4. 10.1016/j.redox.2013.01.006 24024146 PMC3757681

[22] Mizushima N, Komatsu M. Autophagy: renovation of cells and tissues. Cell. 2011; 147:728–41. 10.1016/j.cell.2011.10.026 22078875

[23] Menon MB, Dhamija S. Beclin 1 Phosphorylation - at the Center of Autophagy Regulation. Front Cell Dev Biol. 2018; 6:137. 10.3389/fcell.2018.00137 30370269 PMC6194997

[24] Oikonomou V, Moretti S, Renga G, Galosi C, Borghi M, Pariano M, Puccetti M, Palmerini CA, Amico L, Carotti A, Prezioso L, Spolzino A, Finocchi A, et al. Noncanonical Fungal Autophagy Inhibits Inflammation in Response to IFN-γ via DAPK1. Cell Host Microbe. 2016; 20:744–57. 10.1016/j.chom.2016.10.012 27889463 PMC5161749

[25] Pal S, Tyler JK. Epigenetics and aging. Sci Adv. 2016; 2:e1600584. 10.1126/sciadv.1600584 27482540 PMC4966880

[26] Zhang L, Qi M, Chen J, Zhao J, Li L, Hu J, Jin Y, Liu W. Impaired autophagy triggered by HDAC9 in mesenchymal stem cells accelerates bone mass loss. Stem Cell Res Ther. 2020; 11:269. 10.1186/s13287-020-01785-6 32620134 PMC7333327

[27] Shumaker DK, Dechat T, Kohlmaier A, Adam SA, Bozovsky MR, Erdos MR, Eriksson M, Goldman AE, Khuon S, Collins FS, Jenuwein T, Goldman RD. Mutant nuclear lamin A leads to progressive alterations of epigenetic control in premature aging. Proc Natl Acad Sci U S A. 2006; 103:8703–8. 10.1073/pnas.0602569103 16738054 PMC1472659

[28] Sheikh BN, Phipson B, El-Saafin F, Vanyai HK, Downer NL, Bird MJ, Kueh AJ, May RE, Smyth GK, Voss AK, Thomas T. MOZ (MYST3, KAT6A) inhibits senescence via the INK4A-ARF pathway. Oncogene. 2015; 34:5807–20. 10.1038/onc.2015.33 25772242

[29] Bidon B, Iltis I, Semer M, Nagy Z, Larnicol A, Cribier A, Benkirane M, Coin F, Egly JM, Le May N. XPC is an RNA polymerase II cofactor recruiting ATAC to promoters by interacting with E2F1. Nat Commun. 2018; 9:2610. 10.1038/s41467-018-05010-0 29973595 PMC6031651

[30] Zhang X, Yashiro M, Ren J, Hirakawa K. Histone deacetylase inhibitor, trichostatin A, increases the chemosensitivity of anticancer drugs in gastric cancer cell lines. Oncol Rep. 2006; 16:563–8. 16865256

[31] Davis TR, Trotter KM, Granados RR, Wood HA. Baculovirus expression of alkaline phosphatase as a reporter gene for evaluation of production, glycosylation and secretion. Biotechnology (N Y). 1992; 10:1148–50. 10.1038/nbt1092-1148 1368794

[32] Marthandam Asokan S, Mariappan R, Muthusamy S, Velmurugan BK. Pharmacological benefits of neferine - A comprehensive review. Life Sci. 2018; 199:60–70. 10.1016/j.lfs.2018.02.032 29499283

[33] Li SS, Wu J, Chen LG, Du H, Xu YJ, Wang LJ, Zhang HJ, Zheng XC, Wang LS. Biogenesis of C-glycosyl flavones and profiling of flavonoid glycosides in lotus (Nelumbo nucifera). PLoS One. 2014; 9:e108860. 10.1371/journal.pone.0108860 25279809 PMC4184820

[34] Vermulst M, Bielas JH, Kujoth GC, Ladiges WC, Rabinovitch PS, Prolla TA, Loeb LA. Mitochondrial point mutations do not limit the natural lifespan of mice. Nat Genet. 2007; 39:540–3. 10.1038/ng1988 17334366

[35] Ott C, König J, Höhn A, Jung T, Grune T. Macroautophagy is impaired in old murine brain tissue as well as in senescent human fibroblasts. Redox Biol. 2016; 10:266–73. 10.1016/j.redox.2016.10.015 27825071 PMC5099282

[36] Ito S, Nagata K. Roles of the endoplasmic reticulum-resident, collagen-specific molecular chaperone Hsp47 in vertebrate cells and human disease. J Biol Chem. 2019; 294:2133–41. 10.1074/jbc.TM118.002812 30541925 PMC6369284

[37] Nakamura T, Yamashita M, Ikegami K, Suzuki M, Yanagita M, Kitagaki J, Kitamura M, Murakami S. Autophagy facilitates type I collagen synthesis in periodontal ligament cells. Sci Rep. 2021; 11:1291. 10.1038/s41598-020-80275-4 33446772 PMC7809284

[38] Namba T. BAP31 regulates mitochondrial function via interaction with Tom40 within ER-mitochondria contact sites. Sci Adv. 2019; 5:eaaw1386. 10.1126/sciadv.aaw1386 31206022 PMC6561740

[39] Nogueira-Recalde U, Lorenzo-Gómez I, Blanco FJ, Loza MI, Grassi D, Shirinsky V, Shirinsky I, Lotz M, Robbins PD, Domínguez E, Caramés B. Fibrates as drugs with senolytic and autophagic activity for osteoarthritis therapy. EBioMedicine. 2019; 45:588–605. 10.1016/j.ebiom.2019.06.049 31285188 PMC6642320

[40] Leonard AP, Cameron RB, Speiser JL, Wolf BJ, Peterson YK, Schnellmann RG, Beeson CC, Rohrer B. Quantitative analysis of mitochondrial morphology and membrane potential in living cells using high-content imaging, machine learning, and morphological binning. Biochim Biophys Acta. 2015; 1853:348–60. 10.1016/j.bbamcr.2014.11.002 25447550 PMC4289477

[41] Mou Y, Mukte S, Chai E, Dein J, Li XJ. Analyzing Mitochondrial Transport and Morphology in Human Induced Pluripotent Stem Cell-Derived Neurons in Hereditary Spastic Paraplegia. J Vis Exp. 2020; 60548. 10.3791/60548 32090993 PMC7341675

[42] Wojtala A, Bonora M, Malinska D, Pinton P, Duszynski J, Wieckowski MR. Methods to monitor ROS production by fluorescence microscopy and fluorometry. Methods Enzymol. 2014; 542:243–62. 10.1016/B978-0-12-416618-9.00013-3 24862270

[43] Namba T, Tian F, Chu K, Hwang SY, Yoon KW, Byun S, Hiraki M, Mandinova A, Lee SW. CDIP1-BAP31 complex transduces apoptotic signals from endoplasmic reticulum to mitochondria under endoplasmic reticulum stress. Cell Rep. 2013; 5:331–9. 10.1016/j.celrep.2013.09.020 24139803 PMC3833439

