# Supplementary material for: Lotus germ extract rejuvenates aging fibroblasts via restoration of disrupted proteostasis by the induction of autophagy
Source: Aging (Albany NY). 2022 Sep 26;14(19):7662–91. doi: 10.18632/aging.204303 (PMC9596218; doi:10.18632/aging.204303)
Supplement: Supplementary Figures [file aging-14-204303-s001.pdf]

SUPPLEMENTARY FIGURES

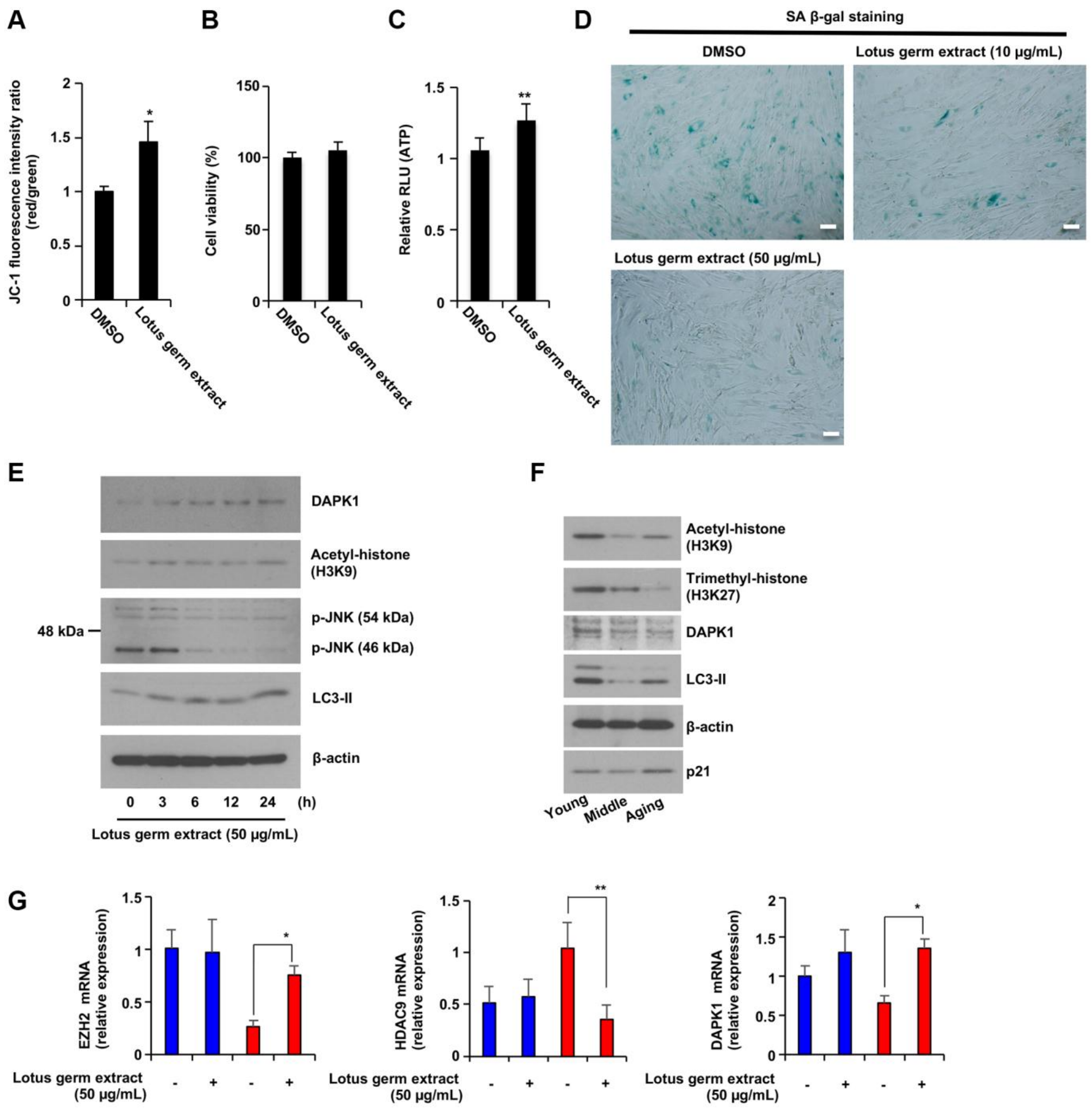

Supplementary Figure 1. IMR90 cells showed the same responses to treatment with lotus germ extract observed in NB1RGB cells. (**A**–**C**) Aging IMR90 cells were treated with DMSO or 50 μg/mL lotus germ extract for 24 h. (**A**) JC-1 activity was determined based on fluorescence intensity. (**B**) Cell viability was determined using an MTT assay. (**C**) ATP levels were determined using a CellTiter-Glo assay. (**D**) Aging IMR90 cells were treated with DMSO or the indicated concentration of lotus germ extract for 3 days, and SA-β-gal activity was measured. (**E**) Aging IMR90 cells were treated with 50 μg/mL lotus germ extract for the indicated times and subjected to immunoblotting using the indicated antibodies. (**F**) IMR90 cells at several passages were subjected to immunoblot assays using the indicated antibodies. (**G**) Young and aging IMR90 cells were treated with DMSO (−) or 50 μg/mL lotus germ extract (+) for 24 h and subjected to qPCR. Data are presented as the mean ± SD of three simultaneous experiments (**A**–**C**, **G**). Each P value was calculated using two-way ANOVA; $^{*}P < 0.05$, $^{**}P < 0.01$.

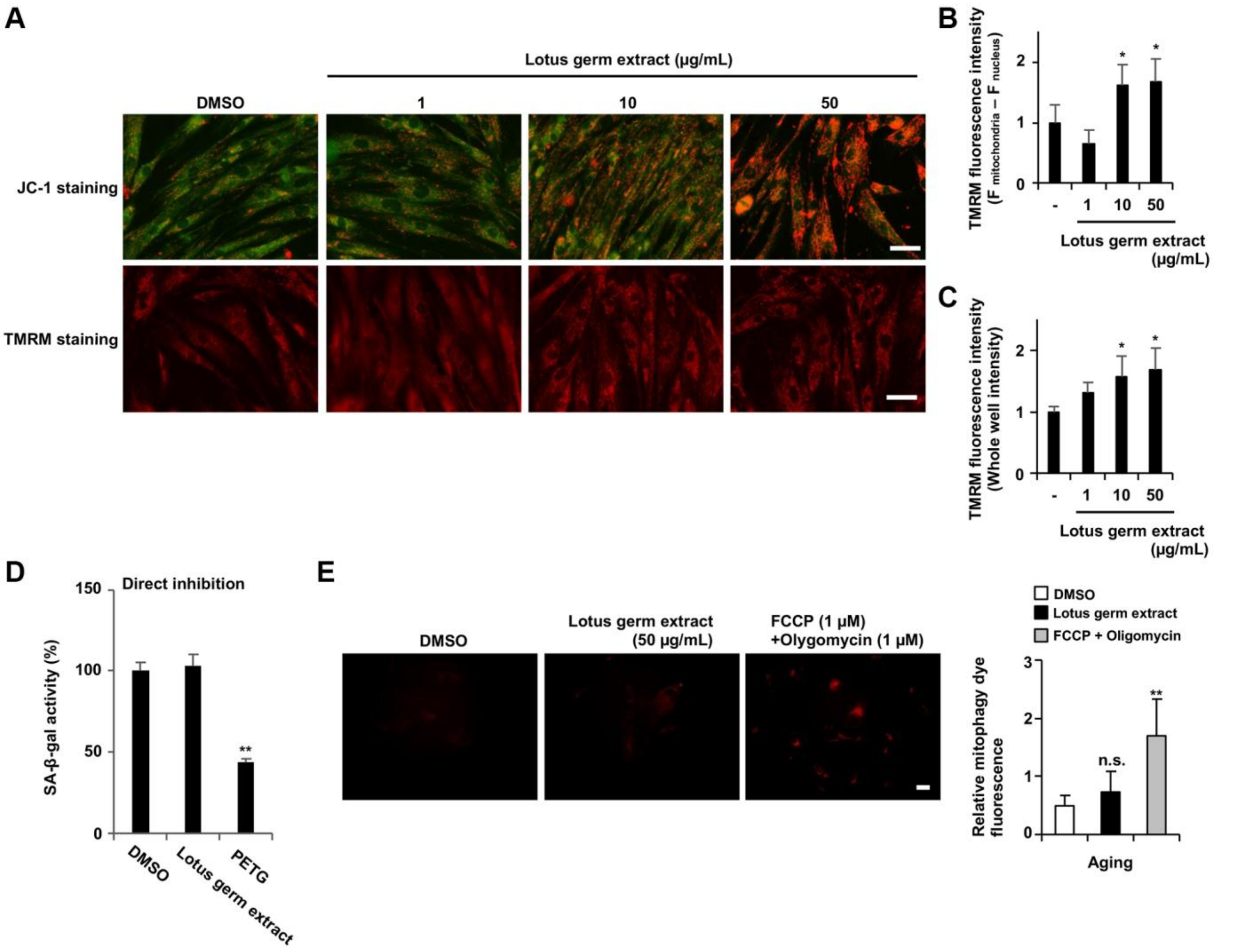

Supplementary Figure 2. Lotus germ extract activated mitochondria function but not induce mitophagy. (**A**–**C**) ΔΨ*m* was determined using the JC-1 or TMRM staining. Treatment with the lotus germ extract increased mitochondrial transmembrane electric potential in aging fibroblasts. The cells were incubated with JC-1 (**A**: upper panel) or TMRM (**A**: under panel, **B**, and **C**) for 20 min and assessed with (**A**) fluorescence microscopy (upper panel: red, JC-1 aggregates: green, JC-1 monomers, under panel: TMRM red fluorescence), (**B**) Mean intensity of TMRM analyze the distribution of the dye between mitochondria and the nucleus or (**C**) microplate reader; TMRM red fluorescence intensity in whole well. (**D**) DMSO, 50 µM lotus germ extract, or 1 mM PETG was added to cell lysates of aging NB1RGB cells, and the SA-β-gal activity was measured. (**E**) Assessment of mitophagy in aging NB1RGB cells with or without 50 µg/mL lotus germ extract or FCCP (1 µM) and oligomycin (1 µM) treatment for 24 h. (**F**, **G**) Lotus germ extract did not affect Beclin1 and LC3 mRNA expression levels. Aging NB1RGB cells were treated with 50 µg/mL Lotus germ extract for 24 h and subjected to real-time quantitative PCR. Data are presented as the mean ± SD of three simultaneous experiments (**B**–**G**). Each *P* value was calculated using two-way ANOVA; n.s.: not significant, $^{**}P < 0.01$.

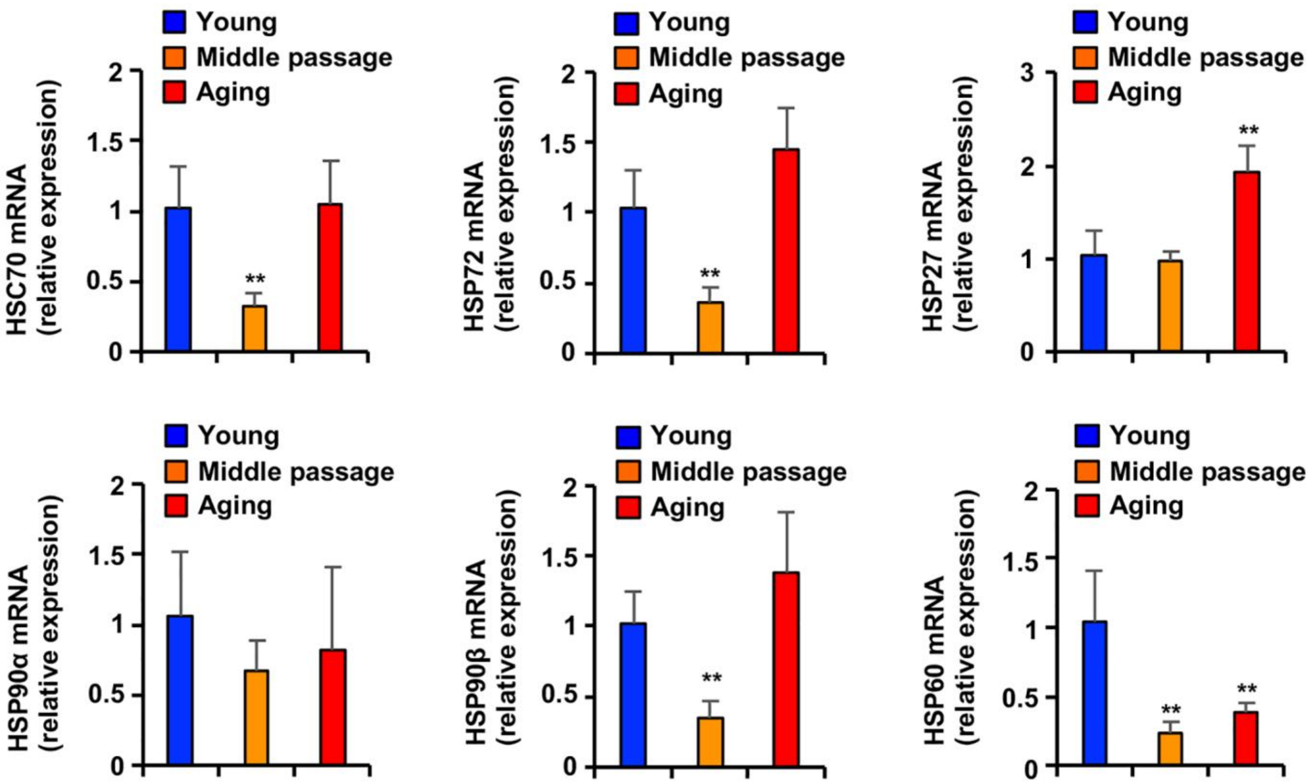

Supplementary Figure 3. Disruption of proteostasis in early phase of aging. Some protein chaperons downregulated in middle passage of fibroblast. Total RNA was extracted from NB1RGB at several passages and subjected to real-time quantitative PCR. Data are presented as the mean ± SD (three different data sets). Each *P* value was calculated using two-way ANOVA; $^{**}P < 0.01$.

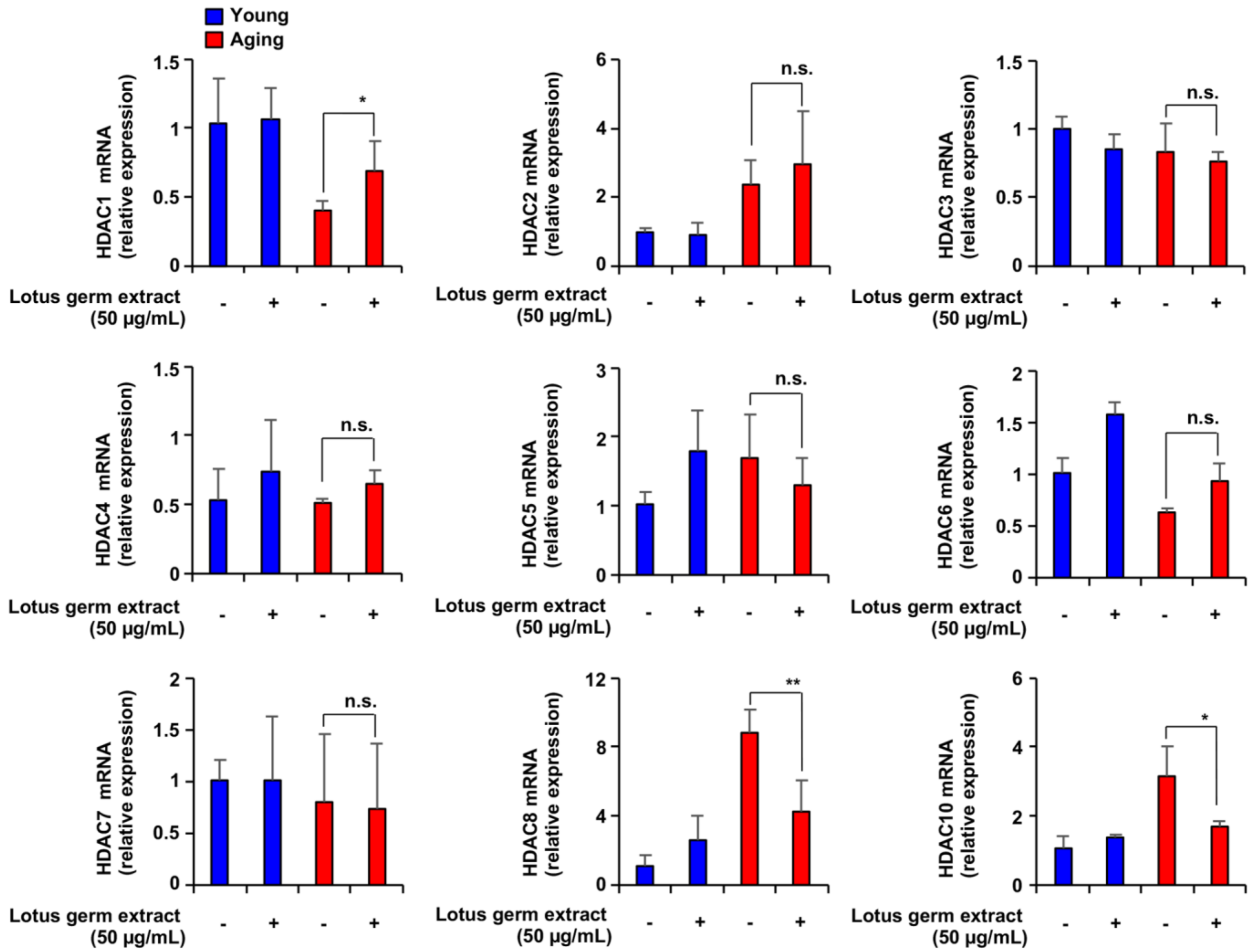

Supplementary Figure 4. Lotus germ extract restored expression of the senescence-dependent modulators HDAC1, HDAC8 and HDAC10. Young and aging NB1RGB cells were treated with DMSO (−) or 50 µg/mL lotus germ extract for 24 h and subjected to qPCR. Data are presented as the mean ± SD of three simultaneous experiments. Each *P* value was calculated using two-way ANOVA; n.s.: not significant, $^{*}P < 0.05$, $^{**}P < 0.01$.

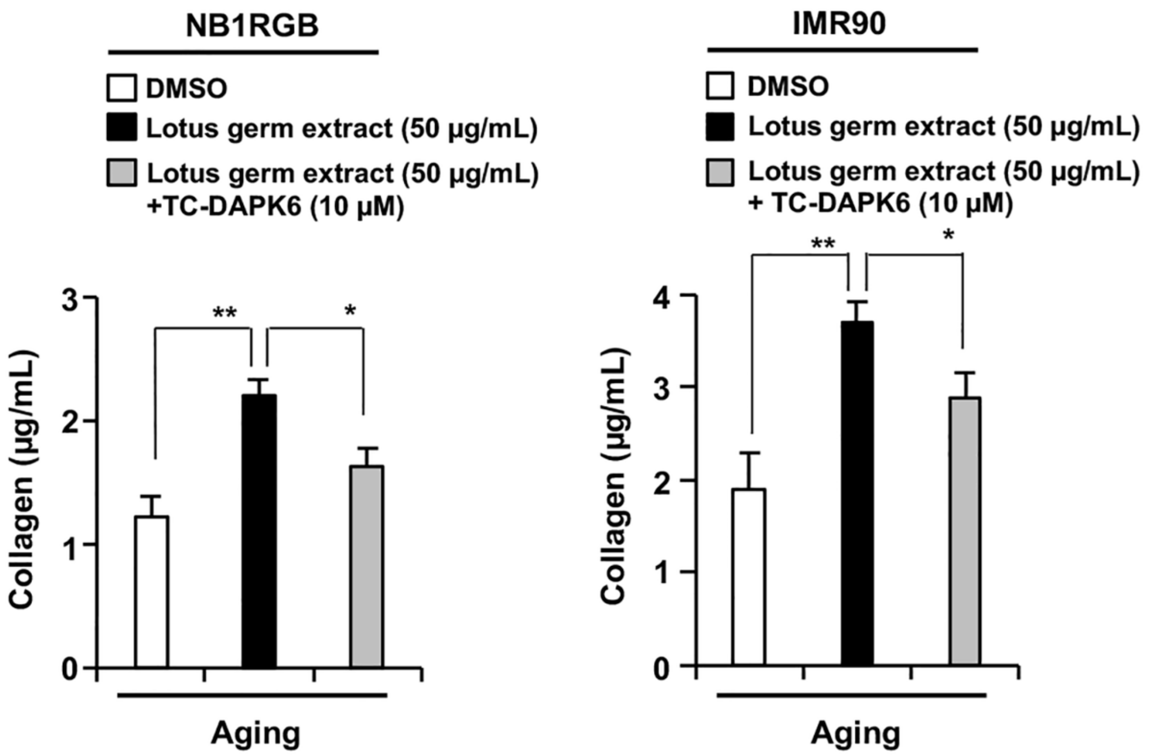

Supplementary Figure 5. DAPK1 inhibition suppressed lotus germ extract-induced collagen production. Aging NB1RGB and IMR90 cells were treated with DMSO (−) or 10 μM TC-DAPK6 and/or 50 μg/mL lotus germ extract for 24 h, and the collagen content was measured. Data are presented as the mean ± SD of three simultaneous experiments. Each *P* value was calculated using two-way ANOVA; $^{*}P < 0.05$, $^{**}P < 0.01$.

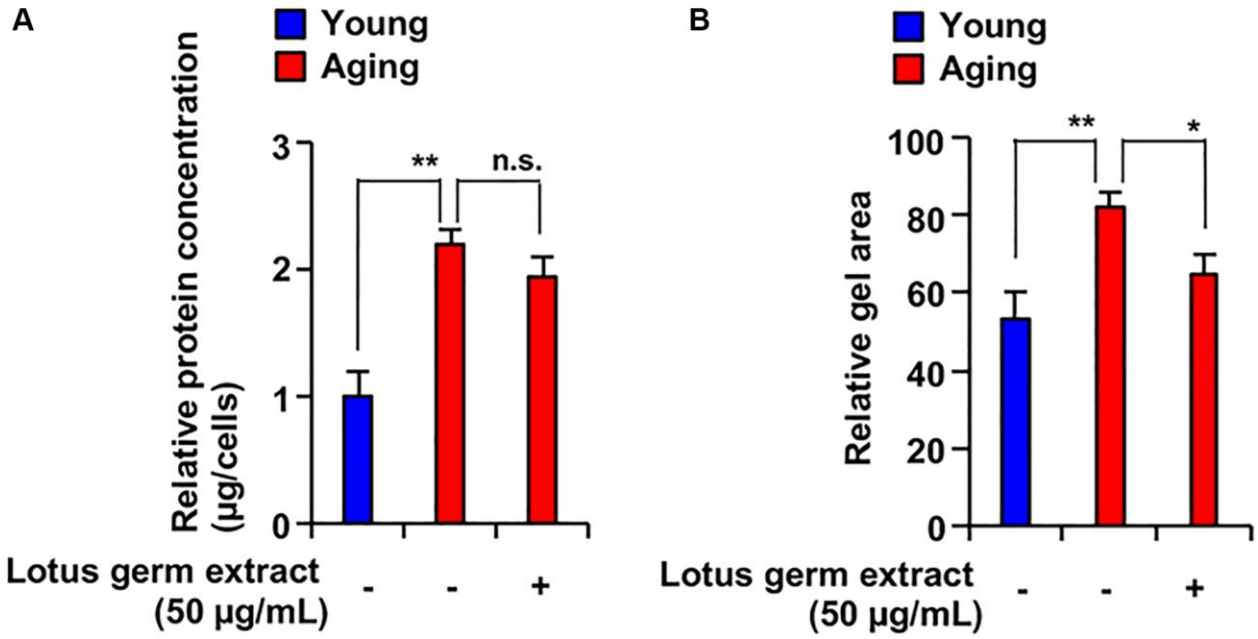

Supplementary Figure 6. Treatment with lotus germ extract restored the lost contractility of aging cells. (**A**) Measurement of protein density per cell in the indicated cells. (**B**) Aging NB1RGB cells were treated with DMSO (−) or t 50 μg/mL lotus germ extract (+) for 72 h. These cells and young cells were cultured in an atelocollagen gel normalized to ensure the same amount of protein per volume, and gel contraction was measured after 24 h. Data are presented as the mean ± SD of three simultaneously performed experiments. Each *P* value was calculated using two-way ANOVA; n.s.: not significant, $^{**}P < 0.01$.

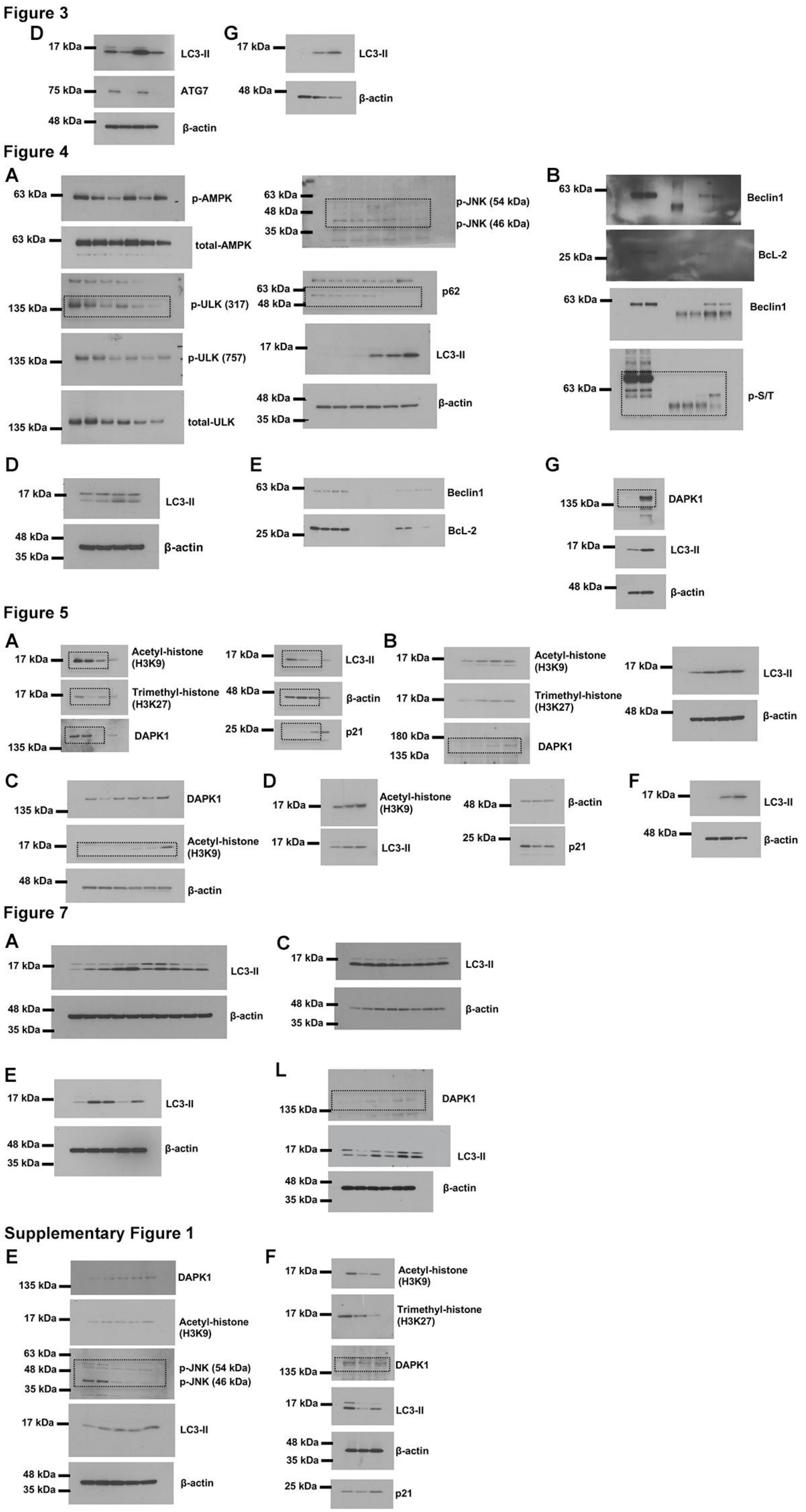

Supplementary Figure 7. The full western blots image.
